# Preventing the transmission of COVID-19 and other coronaviruses in older adults aged 60 years and above living in long-term care: a rapid review

**DOI:** 10.1186/s13643-020-01486-4

**Published:** 2020-09-25

**Authors:** Patricia Rios, Amruta Radhakrishnan, Chantal Williams, Naveeta Ramkissoon, Ba’ Pham, Gordon V. Cormack, Maura R. Grossman, Matthew P. Muller, Sharon E. Straus, Andrea C. Tricco

**Affiliations:** 1 Knowledge Translation Program, Li Ka Shing Knowledge Institute, St. Michael’s Hospital, 209 Victoria Street, East Building, Toronto, Ontario M5B 1W8 Canada; 2 David R. Cheriton School of Computer Science, University of Waterloo, Waterloo, Ontario Canada; 3 Department of Medicine, University of Toronto, Toronto, Ontario Canada; 4 Department of Geriatric Medicine, University of Toronto, Toronto, Ontario Canada; 5 Epidemiology Division and Institute for Health Policy, Management, and Evaluation, Dalla Lana School of Public Health, University of Toronto, Toronto, Ontario, Canada

**Keywords:** COVID-19, Older adults, Long-term care, Clinical guidelines, Knowledge synthesis

## Abstract

**Background:**

The objective of this review was to examine the current guidelines for infection prevention and control (IPAC) of coronavirus disease-19 (COVID-19) or other coronaviruses in adults 60 years or older living in long-term care facilities (LTCF).

**Methods:**

EMBASE, MEDLINE, Cochrane library, pre-print servers, clinical trial registries, and relevant grey literature sources were searched until July 31, 2020, using database searching and an automated method called Continuous Active Learning® (CAL®). All search results were processed using CAL® to identify the most likely relevant citations that were then screened by a single human reviewer. Full-text screening, data abstraction, and quality appraisal were completed by a single reviewer and verified by a second.

**Results:**

Nine clinical practice guidelines (CPGs) were included. The most common recommendation in the CPGs was establishing surveillance and monitoring systems followed by mandating the use of PPE; physically distancing or cohorting residents; environmental cleaning and disinfection; promoting hand and respiratory hygiene among residents, staff, and visitors; and providing sick leave compensation for staff.

**Conclusions:**

Current evidence suggests robust surveillance and monitoring along with support for IPAC initiatives are key to preventing the spread of COVID-19 in LTCF. However, there are significant gaps in the current recommendations especially with regard to the movement of staff between LTCF and their role as possible transmission vectors.

**Systematic review registration:**

PROSPERO CRD42020181993

## Introduction

The current global public health crisis caused by the COVID-19 pandemic has highlighted, among many other things, the critical importance of protecting vulnerable populations from infectious disease. Initial data from the COVID-19 outbreaks in the UK, China, and Italy have shown a significantly increased mortality rate for individuals 60 years of age and above regardless of the presence of comorbid conditions, highlighting the vulnerability of this segment of the population [1–4]. Older adults living in long-term care facilities are especially vulnerable as nosocomial transmission of COVID-19 has been observed in numerous facilities and has contributed to severe outbreaks in healthcare facilities [5, 6]. Thus, it is crucial at this time to ensure that long-term care facilities have adequate access to information to help them prepare for and respond to potential COVID-19 outbreaks.

### Objective and research questions

The overall objective of this rapid review was to examine the current guidelines for the control and prevention of coronavirus disease-19 (COVID-19), Middle East respiratory syndrome (MERS), and severe acute respiratory syndrome (SARS) in adults 60 years or older living in long-term care facilities:

1. What are the infection prevention and control practices for preventing or reducing the transmission of COVID-19, MERS, or SARS in older adults aged 60 years and above living in long-term care?
2. Do the infection prevention and control practices differ for adults aged 60 years and above living in long-term care with severe comorbidities or frailty differ than those without such severe comorbidities or frailty?
3. What are the employment and remuneration policies that may have contributed to the COVID-19 outbreak in adults aged 60 years and above living in long-term care facilities?

## Methods

The conduct of this rapid review was guided by the Rapid Review Guide for Health Policy and Systems [7] published by the World Health Organization (WHO) and reported according to the standards of the PRISMA Checklist [8] (Additional file 1: Appendix 1). A protocol for the review was registered in the PROSPERO database prior to the start of data abstraction (PROSPERO ID: CRD42020181993). A prior review examining infection control guidelines for any form of viral respiratory illness in long-term care leveraged in this work was commissioned by the World Health Organization and posted on a pre-print server (Supplementary File 2) [9]. The results reported here were shared with the Canadian Frailty Network (review commissioner) in the form of a summary report and one-page brief. Our paper has not been published in a peer-reviewed journal.

### Search strategy and selection criteria

In order to accommodate the rapid timeline (10 working days) requested by the review commissioners, a combination of automated and manual search and title/abstract screening methods were used to gather evidence. A comprehensive literature search for the EMBASE database was developed by an experienced librarian and peer-reviewed by a second using the PRESS checklist [10] (Additional file 1: Appendix 2). Grey literature sources (i.e. difficult to locate or unpublished) such as COVID-19-focused evidence gathering services (e.g. EPPI Mapper, COVID-END), as well as guideline producers/repositories (e.g. NICE guidance, ECRI), were hand searched for potentially relevant publications. All other information sources (MEDLINE, Cochrane library, pre-print servers, and clinical trial registries; the full list is available in Additional file 1: Appendix 3) were searched using a supervised machine learning approach, called Continuous Active Learning® (CAL®) [11]. All sources were searched from inception up to July 31, 2020.

CAL® used two methods to gather citations. For sources where the entire archive can be gathered automatically (e.g. MEDLINE), the archive is searched and processed using a priori inclusion criteria established during protocol development. For archives that are only accessible using keyword searches, specific terms drawn from the previously developed literature search (e.g. terms related to COVID-19 and/or long-term care) were applied and the ensuing results were processed using the same inclusion criteria. CAL® identifies titles and abstracts most likely to meet the inclusion criteria based on an iterative process where search results are compared against relevant citations that have previously been identified. The search results from EMBASE and grey literature sources were compared against the CAL® results in order to remove duplicates. The results were then screened against the same inclusion criteria by a human reviewer.

All citations that were included by the title/abstract screening process were then passed on to full-text review. A standardized screening form based on the inclusion criteria was developed and calibrated using a pilot test of 10 full-text articles conducted by the review team. A single human reviewer evaluated the full text of each article using the standardized form, and all excluded full-text articles were screened again by a second, independent reviewer. There was full agreement between both reviewers on the excluded articles.

The inclusion criteria established for this rapid review used the PICOS/T framework as follows:

*➢ Population*: Individuals aged 60 years and above residing in long-term care facilities (e.g. nursing home, long-term care hospital/facility, skilled nursing facility, convalescent home, assisted living facilities). The definition of long-term care that was used is as follows: “Long-term care homes are home-based health care facilities designed for adults who need access to on-site 24-hour nursing care, frequent assistance with activities of daily living (i.e., eating, bathing, toileting, etc.) and monitoring for safety or well-being. They are also known as nursing homes, charitable homes, or municipal homes for the aged” [12, 13].

*➢ Interventions*: Any form of infection prevention and control (IPAC), including but not limited to appropriate ventilation, cohorting equipment, communication, consulting/notifying health professionals, diagnostic testing, environmental cleaning/disinfecting surfaces, droplet precautions, education, access to hand hygiene/hand sanitizer, access to personal protective equipment (PPE; for patients and healthcare providers), policies for visitors, IPAC policies for staff/residents and designated IPAC staff, providing supplies, respiratory hygiene/cough etiquette, smoking cessation, social distancing/isolation/cohorting, surveillance/monitoring/evaluation, antiviral prophylaxis for staff/residents, early mobilization, restrictions on resident movement and transportation, restrictions on visitors, restrictions on travel for healthcare providers, and other long-term care facility staff. Only those measures used to prevent COVID-19, MERS, or SARS were included; measures related to control and prevention of other infections (e.g. vaccination for influenza, oral care to prevent bacterial pneumonia) were excluded. Additionally, interventions related to remuneration/compensation policies for long-term care facility staff, staffing models to maintain staff levels, policies on mixing of staff in long-term care facilities (cohorting), and policies on staff working in more than one long-term care facility were included.

*➢ Comparator*: One of the interventions listed above or no intervention

*➢ Outcomes*: Lab-confirmed respiratory infection [primary outcome], symptoms, secondary transmission (e.g. other patients, healthcare workers), goal concordant care, hospitalization, intensive care unit (ICU) admission, mortality

*➢ Study* designs: clinical practice guidelines (CPGs) and systematic reviews addressing any coronavirus, using the Cochrane definition of a systematic review [14]. Primary human studies of all designs (e.g. experimental studies, quasi-experimental studies, and observational studies, excluding case series) that involved patients with COVID-19 only (not including SARS or MERS) were included.

*➢ Time periods*: All periods of time and duration of follow-up were included.

No other limitations were imposed on the search or study selection process. Both peer-reviewed and pre-print papers were eligible for inclusion, as were papers written in languages other than English.

### Data collection

A standardized data collection form was developed to capture the following information from primary studies: study characteristics (e.g. duration of follow-up, study design, country of conduct, multi-centre vs. single site), patient characteristics (e.g. mean age, age range, comorbidities), intervention details (e.g. type of intervention, duration and frequency of intervention, timing of intervention), comparator details (e.g. comparator intervention, duration and frequency of intervention, timing of intervention), and outcome results (e.g. lab-confirmed respiratory infection [primary outcome], symptoms, secondary transmission, hospitalization, ICU admission, mortality) at the longest duration of follow-up. A separate standardized form was developed to capture relevant information from clinical practice guidelines, including the method used for the guideline, the intended scope of the guideline, and the specific recommendations and level of evidence for each. All data were collected by a single reviewer and verified by a second, independent reviewer.

### Risk of bias appraisal

Standardized and validated appraisal tools were used to assess the included evidence, namely the AGREE-II tool [15] for clinical practice guidelines, the AMSTAR2 tool [16] for systematic reviews, the Cochrane risk of bias (RoB) tool [17] for randomized studies, and the Newcastle-Ottawa Scale [18] for nonrandomized studies. All appraisals were completed by a single reviewer and verified by a second, independent reviewer.

### Synthesis

As this review was conducted over a very short timeline and incorporated diverse evidence sources, no formal statistical or qualitative analysis was planned. Data from the included studies are summarized narratively and in summary tables and detailed tables of results.

## Results

### Literature search

**Fig. 1:**
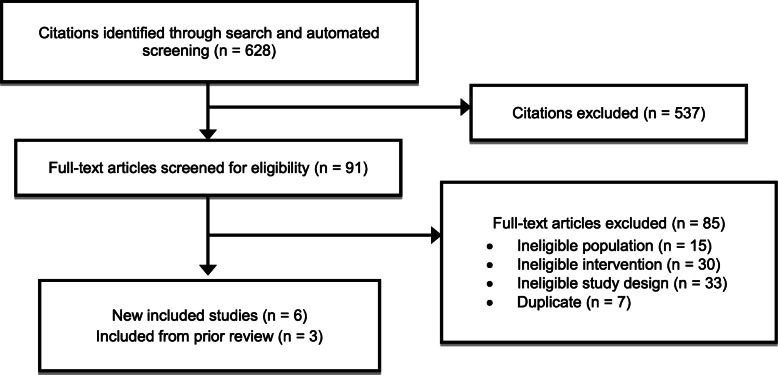
Study flow diagram

The manual searches and automated search and screening process returned a combined total of 628 titles and abstracts for further evaluation. A total of 537 title and abstract citations were then excluded, and 91 articles were passed to full-text screening. A further 85 articles were excluded during full-text screening leaving six included policy guidelines [19–24]. These guidelines were combined with three clinical practice guidelines (CPGs) [25–27] found during an earlier review [9] for a total of 9 articles included in this review (Fig. 1).

### Characteristics of included articles

All of the included guidelines were published in the year 2020 in the USA (*n* = 5), South Africa (*n* = 1), the UK (*n* = 1), and Canada (*n* = 1). The nine included CPGs were published by government agencies [23, 25, 27] (*n* = 3), medical associations (*n* = 3) [19, 22, 24], non-profit research trusts [20, 26] (*n* = 2), or international health organizations (*n* = 1) [21]; target audiences included administration, staff, residents, and visitors of long-term care facilities of any type (Additional file 1: Appendix 4).

The nine included clinical practice guidelines were of very low quality as they reported very few items across the assessment domains (Table 1; full results in Additional file 1: Appendix 5). For the scope and purpose domain, the clinical practice guidelines reported two to six of the relevant details out of 12. For the stakeholder domain, the guidelines reported one to six of the relevant details out of 11. For the rigour of the development domain, only two guidelines reported one relevant detail out of 35 items. For the clarity of the presentation domain, the guidelines reported two to seven of the relevant details out of eight. For the applicability domain, five guidelines each reported one of the relevant details out of 13. For the editorial independence domain, the guidelines reported 0 to two of the relevant details out of six.

**Table 1:** Summary AGREE-II scores

	AGS, 2020 [19]	CDC, 2020 [25]	ECRI, 2020a [26]	ECRI, 2020b [20]	Geffen, 2020	HPS, 2020 [23]	Lester, 2020 [24]	MOH, 2020 [27]	WHO, 2020 [21]
Domain 1: Scope and purpose (12 points in total)									
Item 1: Objectives	3	2	1	1	1	2	2	2	1
Item 2: Questions	1	1	1	1	0	0	0	1	0
Item 3: Population	2	1	2	1	1	2	2	1	2
Totals	6	4	4	3	2	4	4	4	3
Domain 2: Stakeholder involvement (11 points in total)									
Item 4: Group membership	1	0	0	0	0	1	3	0	0
Item 5: Target population preferences and views	0	0	0	0	0	0	2	0	0
Item 6: Target users	1	1	0	1	2	1	1	1	1
Totals	2	1	0	1	2	2	6	1	1
Domain 3: Rigour of development (35 points in total)									
Item 7: Search methods	0	0	0	0	0	0	1	0	0
Item 8: Evidence selection criteria	0	0	0	0	0	0	0	0	0
Item 9: Strengths and limitations of the evidence	0	0	0	0	0	0	0	0	0
Item 10: Formulation of recommendations	0	0	0	1	0	0	0	0	0
Item 11: Consideration of benefits and harms	0	0	0	0	0	0	0	0	0
Item 12: Link between recommendations and evidence	0	0	0	0	0	0	0	0	0
Item 13: External review	0	0	0	0	0	0	0	0	0
Item 14: Updating procedure	0	0	0	0	1	0	0	0	0
Totals	0	0	0	1	1	0	1	0	0
Domain 4: Clarity of presentation (8 points in total)									
Item 15: Specific and unambiguous recommendations	3	3	3	1	2	2	3	2	1
Item 16: Management options	0	0	2	1	1	1	1	1	1
Item 17: Identifiable key recommendations	0	1	2	0	2	2	2	1	1
Totals	3	4	7	2	5	5	6	4	3
Domain 5: Applicability (13 points in total)									
Item 18: Facilitators and barriers to application	0	0	0	0	0	0	0	0	0
Item 19: Implementation advice/tools	0	1	0	1	1	1	0	1	0
Item 20: Resource implications	0	0	0	0	0	0	0	0	0
Item 21: Monitoring/auditing criteria	0	0	0	0	0	0	0	0	0
Totals	0	1	0	1	1	1	0	1	0
Domain 6: Editorial independence (6 points in total)									
Item 22: Funding body	1	1	1	0	0	1	2	1	1
Item 23: Competing interests	1	0	0	0	0	0	0	0	0
Totals	2	1	1	0	0	1	2	1	1

**Table 2:** Summary of recommendations from included clinical practice guidelines

Recommendations	AGS, 2020 [19]	CDC, 2020 [25]	ECRI, 2020a [26]	ECRI, 2020b [20]	Geffen, 2020	HPS, 2020 [23]	Lester, 2020 [24]	MOH, 2020 [27]	WHO, 2020 [21]
Cohorting equipment			X						
Communication				X		X	X		X
Consulting/notifying health professionals	X		X					X	
Diagnostic testing								X	
Disinfecting surfaces		X	X		X	X		X	X
Droplet precautions								X	X
Education	X	X		X					X
Hand hygiene		X	X		X	X		X	
Personal protective equipment		X	X		X	X	X	X	X
Policies for visitors		X			X			X	
Provide supplies	X	X		X	X				
Respiratory hygiene/cough etiquette		X		X	X	X		X	
Social distancing/isolation/cohorting	X	X	X		X	X	X		X
Staffing policies	X	X			X	X		X	
Surveillance/monitoring/evaluation	X	X		X	X	X	X	X	X

### CPG recommendations

The included CPGs recommended a variety of interventions that were summarized into 16 general categories (Table 2; Additional file 1: Appendices 6 and 7). The most common recommendation was establishing surveillance, monitoring, and evaluation of symptoms/illness among staff and residents, present in eight of the nine included CPGs. The next most frequent recommendations were included in five or more CPGs: mandating the use of appropriate personal protective equipment (PPE) for staff, residents, and/or visitors; employing social distancing or isolation measures to prevent the spread of COVID-19 (e.g. serving resident meals in individual rooms, cancelling group activities in the facility) and/or cohorting (isolating) patients with confirmed or suspected COVID-19; routine or increased disinfection of surfaces in the facility; promoting and enforcing hand-hygiene measures among staff, residents, and/or visitors; promoting and enforcing respiratory hygiene measures among staff, residents, and/or visitors; and implementing staffing policies to promote and enforce mandatory sick leave for staff with symptoms or suspected COVID-19 and/or ensure adequate compensation for staff on sick leave as well as policies to restrict the movement of staff within or between facilities. Other recommendations included in two to four CPGs were ensuring appropriate communication between long-term care facilities and local/regional health authorities; educating staff and/or residents on appropriate infection control, hand, or respiratory hygiene; ensuring adequate supplies of PPE, medications, and other medical equipment (e.g. ventilators) to manage COVID-19 outbreaks; consulting with and notifying relevant health professionals to deal with COVID-19 cases; policies restricting visitor hours or limiting to “essential” visitors only; and mandating the use of droplet precautions (including appropriate PPE) when treating any patient suspected or confirmed to have COVID-19. Finally, the following recommendations were only included in one CPG each: cohorting certain equipment to only be used with COVID-19 patients and testing all symptomatic staff and/or residents for COVID-19.

## Discussion

This rapid review aimed to address the question of what are effective IPAC measures for COVID-19 that can be used in long-term care. It is especially relevant given the current COVID-19 outbreaks in such facilities globally including the UK and Canada. A comprehensive literature search that included electronic sources, grey literature sites, and references from a prior review produced nine clinical practice guidelines dealing specifically with the control and management of COVID-19 and SARS among older adults in long-term care. None of the included articles addressed MERS or infection control and prevention for frail older adults or those with significant comorbidity. Only five of the included CPGs addressed how compensation or leave policies or policies restricting staff to one work location could affect the transmission of COVID-19 in long-term care facilities.

Among the nine included CPGs, the most commonly recommended strategy was establishing surveillance, monitoring, and evaluation within long-term care facilities, followed by mandating the use of PPE, employing physical distancing/isolation or cohorting measures among residents of a facility, disinfecting surfaces, promoting hand hygiene, promoting respiratory hygiene/cough etiquette, implementing policies regarding staff sick leave or restricting staff movement, establishing clear communication means and consulting with or notifying relevant healthcare authorities and ensuring appropriate action is taken, educating staff and/or residents on infection control and hygiene, ensuring adequate supplies for facilities, mandating droplet precautions, and policies restricting visitors to long-term care. It should be noted however that many of the included guidelines were prepared as rapid responses to an urgent situation and thus had to forgo more formal guideline development methods in favour of expedience. In this particular case, low appraisal scores should not necessarily be indicative of untrustworthy recommendations. However, as these recommendations have not gone through the rigorous process of formal guideline development neither should they be taken at face value as they may not reflect the best available evidence.

The gaps in evidence that this review has identified are particularly important. For example, much has been written about how personal support workers or care home providers are underpaid and often have to work across multiple institutions [3]. However, there is a paucity of literature on how compensation models for healthcare workers in long-term care could impact outbreaks. Moreover, while the identified guidelines recommend various strategies including adequate PPE use, one of the common global challenges has been supply chain shortages. This issue highlights that practical or technological barriers to implementing guideline recommendations must also be considered. Furthermore, the majority of existing IPAC guidelines relevant to long-term care facilities focus on managing viral outbreaks caused by influenza and emphasize the use of vaccination or chemoprophylaxis, both of which are options not currently available in the control of COVID-19 or other coronaviruses [9]. The most up-to-date knowledge base for prevention and control of COVID-19 is likely not in guidelines specifically tailored to long-term care but rather acute or hospital settings, highlighting the fact that long-term care facilities need substantial additional support and resources to effectively tackle this crisis.

There are several limitations to the review methods employed here primarily the lack of duplicate screening and abstraction. However, these methods were selected to tailor our approach to our knowledge-user needs and satisfy the urgent need to provide timely results. There is also a chance that our literature search missed guidance documents from various state and provincial authorities that address IPAC in other types of healthcare facilities (e.g. hospitals or acute care) that could be successfully applied to long-term care facilities. However, we were unable to perform an exhaustive grey literature search of guideline repositories and agency websites due to the timelines imposed on this review.

The current guidelines on preventing transmission of COVID-19 in long-term care facilities seem to suggest that robust surveillance and monitoring programs, accompanied with environmental cleaning measures and supporting the use of PPE, hand/respiratory hygiene, and physical distancing, are the ideal approach to protect older adults. However, there are significant gaps in the current recommendations, especially related to the movement of staff between long-term care facilities, as well as an overall lack of guidelines specific to managing highly virulent outbreaks in long-term care facilities.

## Supplementary information

**Additional file 1:.** Appendices. Appendix 1 – PRISMA Checklist. Appendix 2 – Embase Search Strategy. Appendix 3 – Grey Literature Sources. Appendix 4 – Clinical Practice Guideline Characteristics. Appendix 5 – Detailed Quality Appraisal Results for Clinical Practice Guidelines. Appendix 6 – Clinical Practice Guideline Results. Appendix 7 – CPG Coding Summary and Supporting Text

**Additional file 2:.** Guidelines for preventing respiratory illness in older adults aged 60 years and above living in long-term care: A rapid review of clinical practice guidelines (pre-print report)

## Data Availability

The full dataset is available from the corresponding author upon reasonable request.

## Abbreviations

CAL: Continuous Active Learning
COVID-19: Coronavirus disease-19
CPG: Clinical practice guideline
ICU: Intensive care unit
MERS: Middle East respiratory syndrome
PPE: Personal protective equipment
PRESS: Peer Review of Electronic Search Strategies
SARS: Severe acute respiratory syndrome
WHO: World Health Organization
