# Supplementary material for: Preventing the transmission of COVID-19 and other coronaviruses in older adults aged 60 years and above living in long-term care: a rapid review
Source: Syst Rev. 2020 Sep 25;9:218. doi: 10.1186/s13643-020-01486-4 (PMC7517751; doi:10.1186/s13643-020-01486-4)
Supplement: Supplementary file 1 — Additional file 1:. Appendices. Appendix 1 – PRISMA Checklist. Appendix 2 – Embase Search Strategy. Appendix 3 – Grey Literature Sources. Appendix 4 – Clinical Practice Guideline Characteristics. Appendix 5 – Detailed Quality Appraisal Results for Clinical Practice Guidelines. Appendix 6 – Clinical Practice Guideline Results. Appendix 7 – CPG Coding Summary and Supporting Text [file 13643_2020_1486_MOESM1_ESM.docx]

# APPENDIX 1 – PRISMA Checklist

| **Section/topic** | **#** | **Checklist item** | **Reported on page #** |
| --- | --- | --- | --- |
| **TITLE** | | |  |
| Title | 1 | Identify the report as a systematic review, meta-analysis, or both. | 1 |
| **ABSTRACT** | | |  |
| Structured summary | 2 | Provide a structured summary including, as applicable: background; objectives; data sources; study eligibility criteria, participants, and interventions; study appraisal and synthesis methods; results; limitations; conclusions and implications of key findings; systematic review registration number. | 2 |
| **INTRODUCTION** | | |  |
| Rationale | 3 | Describe the rationale for the review in the context of what is already known. | 4 |
| Objectives | 4 | Provide an explicit statement of questions being addressed with reference to participants, interventions, comparisons, outcomes, and study design (PICOS). | 4 |
| **METHODS** | | |  |
| Protocol and registration | 5 | Indicate if a review protocol exists, if and where it can be accessed (e.g., Web address), and, if available, provide registration information including registration number. | 5 |
| Eligibility criteria | 6 | Specify study characteristics (e.g., PICOS, length of follow-up) and report characteristics (e.g., years considered, language, publication status) used as criteria for eligibility, giving rationale. | 6-8 |
| Information sources | 7 | Describe all information sources (e.g., databases with dates of coverage, contact with study authors to identify additional studies) in the search and date last searched. | 5-6 |
| Search | 8 | Present full electronic search strategy for at least one database, including any limits used, such that it could be repeated. | Appendix 2 |
| Study selection | 9 | State the process for selecting studies (i.e., screening, eligibility, included in systematic review, and, if applicable, included in the meta-analysis). | 6 |
| Data collection process | 10 | Describe method of data extraction from reports (e.g., piloted forms, independently, in duplicate) and any processes for obtaining and confirming data from investigators. | 8 |
| Data items | 11 | List and define all variables for which data were sought (e.g., PICOS, funding sources) and any assumptions and simplifications made. | 8 |
| Risk of bias in individual studies | 12 | Describe methods used for assessing risk of bias of individual studies (including specification of whether this was done at the study or outcome level), and how this information is to be used in any data synthesis. | 9 |
| Summary measures | 13 | State the principal summary measures (e.g., risk ratio, difference in means). | N/A |
| Synthesis of results | 14 | Describe the methods of handling data and combining results of studies, if done, including measures of consistency (e.g., I^2^) for each meta-analysis. | N/A |
| Risk of bias across studies | 15 | Specify any assessment of risk of bias that may affect the cumulative evidence (e.g., publication bias, selective reporting within studies). | N/A |
| Additional analyses | 16 | Describe methods of additional analyses (e.g., sensitivity or subgroup analyses, meta-regression), if done, indicating which were pre-specified. | N/A |
| RESULTS |  |  |  |
| Study selection | 17 | Give numbers of studies screened, assessed for eligibility, and included in the review, with reasons for exclusions at each stage, ideally with a flow diagram. | 9, Figure 1 |
| Study characteristics | 18 | For each study, present characteristics for which data were extracted (e.g., study size, PICOS, follow-up period) and provide the citations. | 9-10, Appendices 4-5 |
| Risk of bias within studies | 19 | Present data on risk of bias of each study and, if available, any outcome level assessment (see item 12). | 10-11, Appendix 6 |
| Results of individual studies | 20 | For all outcomes considered (benefits or harms), present, for each study: (a) simple summary data for each intervention group (b) effect estimates and confidence intervals, ideally with a forest plot. | N/A |
| Synthesis of results | 21 | Present the main results of the review. If meta-analyses are done, include for each, confidence intervals and measures of consistency | 11-12 |
| Risk of bias across studies | 22 | Present results of any assessment of risk of bias across studies (see Item 15). | N/A |
| Additional analysis | 23 | Give results of additional analyses, if done (e.g., sensitivity or subgroup analyses, meta-regression [see Item 16]). | N/A |
| DISCUSSION |  |  |  |
| Summary of evidence | 24 | Summarize the main findings including the strength of evidence for each main outcome; consider their relevance to key groups (e.g., healthcare providers, users, and policy makers). | 13-14 |
| Limitations | 25 | Discuss limitations at study and outcome level (e.g., risk of bias), and at review-level (e.g., incomplete retrieval of identified research, reporting bias). | 14 |
| Conclusions | 26 | Provide a general interpretation of the results in the context of other evidence, and implications for future research. | 15 |
| FUNDING |  |  |  |
| Funding | 27 | Describe sources of funding for the systematic review and other support (e.g., supply of data); role of funders for the systematic review. | 16-17 |

#

# APPENDIX 2 – Embase Search Strategy

Database: Embase <1974 to 2020 April 15>

Search Strategy:

--------------------------------------------------------------------------------

1 exp coronaviridae/ or exp Coronaviridae infection/ or exp Coronavirus infection/ or SARS coronavirus/

2 ((wuhan or hubei or huanan) and (severe acute respiratory or pneumonia* or virus*) and outbreak*).mp.

3 (coronavir* or "corona virus*" or "coronavirus pneumonia" or betacoronavir* or COVID or COVID-19).mp.

4 ("nCoV" or "cov 2" or cov2 or 2019ncov or 2019-nCoV or "2019 ncov" or "2019-ncov" or "2019 novel cov" or "2019 ncov disease*" or "2019 novel coronavirus*").mp.

5 ("severe acute respiratory syndrome coronavirus*" or "wuhan virus*" or "sars cov 2 mers" or "middle east respiratory syndrome*" or "Severe Acute Respiratory" or SARS or SARS-CoV or SARScov2 or MERS-CoV).mp.

6 or/1-5

7 exp communicable disease control/ or exp "prevention and control"/

8 contact examination/

9 exp protective equipment/ or exp surgical attire/

10 exp hygiene/ or exp hand washing/

11 patient isolation/ or contact examination/

12 instrument sterilization/ or exp disinfection/ or decontamination/

13 bleaching agent/

14 ("infection control" or "virus control" or "disease control" or prevent* or handwash* or "hand wash*" or quarant* or isolat* or steril* or disinfect* or fumigat* or decontaminat* or resanitiz* or resanitis* or desaniti* or contaminat* or antisept* or biocid* or steriliz* or sanitize* or bleach* or hypochlor* or ozon* or ultraviolet or UV or "contract tracing" or "disease notification").mp.

15 ("protective equipment" or "protective cloth*" or "protective product*" or "protective gear" or PPE or PPEs or mask* or facemask* or half-mask* or facepiece* or n95* or n99* or shield* or faceshield* or "Particulate filter*" or "gas filter*" or glov* or gown or gowns or "space suits" or "respiratory protect*" or visor or "eye protect* " or "eye spectacle* " or "hand protect* " or "hand wash*" or "handwash*" or google or goggles or "head cover* " or "shoe cover*" or respirator* or ventilator*).mp.

16 (restrict* adj3 (resident* or patient* or visit* or family or travel* or staff or provider* or employee*)).mp.

17 ((respiratory or cough or hand) adj2 (hygiene or etiquette)).mp.

18 exp ventilator/

19 or/7-18

20 6 and 19

21 nursing home/ or home for the aged/ or assisted living facility/

22 ((elder* or senior or nursing or aged or "old age" or "old people" or "old person*" or "long-term care" or "LTC" or "long term care") adj2 (home or homes or hous* or residenc* or facilit* or hospital*)).mp.

23 ("convalescence hom*" or "convalescence hospital*" or "extended care facility*" or "charitable hom*" or " home based health care facilit*").mp.

24 exp long term care/

25 or/21-24

26 20 and 25

# APPENDIX 3 – Grey Literature Sources

- US National Library of Medicine
  - <https://clinicaltrials.gov/>
- WHO International Clinical Trials Registry Platform
  - <https://www.who.int/ictrp/en/>
- COVID-19 Evidence Alerts
  - <https://plus.mcmaster.ca/COVID-19/>
- McMaster Evidence Forum – Covid-19 Evidence Network to support Decision-making (COVID-END)
  - <https://www.mcmasterforum.org/networks/covidend/lets-collaborate/our-guide-to-covid-19-evidence-sources>
- Joanna Briggs Institute – COVID-19 Special Collection
  - <https://jbi.global/ebp/covid-19>
- Evidence aid – Coronavirus (COVID-19): Evidence Collection
  - <https://www.evidenceaid.org/coronavirus-covid-19-evidence-collection/>
- National Collaborating Centre for Methods and Tools – COVID-19 Rapid Evidence Reviews
  - <https://www.nccmt.ca/knowledge-repositories/covid-19-evidence-reviews>
- PROSPERO – International prospective register of systematic reviews
  - <https://www.crd.york.ac.uk/prospero/>
- EPPI Mapper – COVID-19: living map of the evidence
  - <http://eppi.ioe.ac.uk/COVID19_MAP/covid_map_v6.html>
- NIPH systematic and living map on COVID-19 evidence
  - <https://www.nornesk.no/forskningskart/NIPH_mainMap.html>
- ECRI Institute – COVID-19 Resource Center
  - <https://www.ecri.org/coronavirus-covid-19-outbreak-preparedness-center/>
- WHO Country & Technical Guidance - Coronavirus disease (COVID-19)
  - <https://www.who.int/emergencies/diseases/novel-coronavirus-2019/technical-guidance>
- National Institute for Health and Care Excellence (NICE) – COVID-19 guidance
  - <https://www.nice.org.uk/covid-19>
- BIGG International database of GRADE guidelines
  - <https://sites.bvsalud.org/bigg/en/biblio/>
- MAGICapp – Guidelines database
  - [https://app.magicapp.org/app#/guidelines](https://app.magicapp.org/app%23/guidelines)
- Guidelines International Network (G-I-N) – COVID-19 Resources
  - https://g-i-n.net/covid-19
- National COVID-19 Evidence Taskforce (Australia)
  - [https://covid19evidence.net.au/#living-guidelines](https://covid19evidence.net.au/%23living-guidelines)

#


# APPENDIX 4 – Clinical Practice Guideline Characteristics

| Author, Year  Country | Evidence Collection | Guideline Development | Participants | Guideline Scope |
| --- | --- | --- | --- | --- |
| AGS, 2020  USA | NR | Recommendations by the American Geriatrics Society based on guidance set forth in peer-reviewed articles and editorials, as well as ongoing and updated guidance from the Centers for Medicare and Medicaid Services (CMS), the Centers for Disease Control and Prevention (CDC), and other key agencies. | executive officers of the AGS Board of Directors | recommendations to guide federal, state, and local governments when making decisions about how best to care for patients with COVID-19 in nursing homes (NHs) and other long-term care facilities (LTCFs) |
| CDC, 2020  USA | NR | NR | CDC | Preventing COVID in nursing homes.  Recommendations are specific for nursing homes, including skilled nursing facilities. Much of this information could be applied in assisted living facilities. |
| ECRI, 2020a  USA | NR | NR | ECRI | Advance preparation to limit the spread of infection |
| ECRI, 2020b  USA | NR | NR | ECRI | Infection control and prevention procedures and procedures during equipment servicing |
| Geffen, 2020  South Africa | NR | NR | NR | This document has four sections: 1) infection prevention, 2) testing and screening, 3) managing infection/outbreak, and 4) broader policies and planning |
| HPS, 2020  United Kingdom | NR | NR | NR | This guidance is to support those working in care home settings to give advice to their staff and users of their services about COVID-19. |
| Lester, 2020  USA | Literature review through PubMed was conducted and review of studies at ClinicalTrials.gov | The consensus statements presented here have been formulated by the authors who had experience with outbreaks of COVID-19 as the SNF community needed to rapidly adapt to the dynamic changes that occurred in these healthcare facilities during this unprecedented pandemic. | The authors are actively working Certified Medical Directors, are Board Members of the New York Medical Directors Association, and serve as Medical Directors in Long Island, New Rochelle, and Rochester. | We describe expert consensus policies for SNFs to prepare for and manage COVID-19. |
| MOH, 2020  Canada | NR | NR | NR | Screening, infection control for COVID-19 in LTC |
| WHO, 2020  NR | NR | NR | NR | The objective of this document is to provide guidance on IPC in LTCFs in the context of COVID-19 to 1) prevent COVID-19-virus from entering the facility, 2) prevent COVID-19 from spreading within the facility, and 3) prevent COVID-19 from spreading to outside the facility |

# APPENDIX 5 – Detailed Quality Appraisal Results for Clinical Practice Guidelines

| CHECKLIST ITEM AND DESCRIPTION | REPORTING CRITERIA |
| --- | --- |
| Article: AGS, 2020 |  |
| DOMAIN 1: SCOPE AND PURPOSE |  |
| 1. OBJECTIVES  Report the overall objective(s) of the guideline. The expected health benefits from the guideline are to be specific to the clinical problem or health topic. | Health intent(s) (i.e., prevention, screening, diagnosis, treatment, etc.)  Expected benefit(s) or outcome(s)  Target(s) (e.g., patient population, society) |
| 2. QUESTIONS  Report the health question(s) covered by the guideline, particularly for the key recommendations | Intervention(s) or exposure(s)  Comparisons (if appropriate)  Outcome(s)  Health care setting or context |
| 3. POPULATION  Describe the population (i.e., patients, public, etc.) to whom the guideline is meant to apply. | Target population, sex and age  Clinical condition (if relevant)  Severity/stage of disease (if relevant)  Comorbidities (if relevant)  Excluded populations (if relevant) |
| DOMAIN 2: STAKEHOLDER INVOLVEMENT |  |
| 4. GROUP MEMBERSHIP  Report all individuals who were involved in the development process. This may include members of the steering group, the research team involved in selecting and reviewing/rating the evidence and individuals involved in formulating the final recommendations | Name of participant  Discipline/content expertise (e.g., neurosurgeon, methodologist)  Institution (e.g., St. Peter’s hospital)  Geographical location (e.g., Seattle, WA)  A description of the member’s role in the guideline development group |
| 5. TARGET POPULATION PREFERENCES AND VIEWS  Report how the views and preferences of the target population were sought/considered and what the resulting outcomes were. | Statement of type of strategy used to capture patients’/publics’ views and preferences (e.g., participation in the guideline development group, literature review of values and preferences)  Methods by which preferences and views were sought (e.g., evidence from literature, surveys, focus groups)  Outcomes/information gathered on patient/public information  How the information gathered was used to inform the guideline development process and/or formation of the recommendations |
| 6. TARGET USERS  Report the target (or intended) users of the guideline | The intended guideline audience (e.g. specialists, family physicians, patients, clinical or institutional leaders/administrators)  How the guideline may be used by its target audience (e.g., to inform clinical decisions, to inform policy, to inform standards of care) |
| DOMAIN 3: RIGOUR OF DEVELOPMENT |  |
| 7. SEARCH METHODS  Report details of the strategy used to search for evidence. | Named electronic database(s) or evidence source(s) where the search was performed (e.g., MEDLINE, EMBASE, PsychINFO, CINAHL)  Time periods searched (e.g., January 1, 2004 to March 31, 2008)  Search terms used (e.g., text words, indexing terms, subheadings)  Full search strategy included (e.g., possibly located in appendix) |
| 8. EVIDENCE SELECTION CRITERIA  Report the criteria used to select (i.e., include and exclude) the evidence. Provide rationale, where appropriate. | Target population (patient, public, etc.) characteristics  Study design  Comparisons (if relevant)  Outcomes  Language (if relevant)  Context (if relevant) |
| 9. STRENGTHS & LIMITATIONS OF THE EVIDENCE  Describe the strengths and limitations of the evidence. Consider from the perspective of the individual studies and the body of evidence aggregated across all the studies. Tools exist that can facilitate the reporting of this concept. | Study design(s) included in body of evidence  Study methodology limitations (sampling, blinding, allocation concealment, analytical methods)  Appropriateness/relevance of primary and secondary outcomes considered  Consistency of results across studies  Direction of results across studies  Magnitude of benefit versus magnitude of harm  Applicability to practice context |
| 10. FORMULATION OF RECOMMENDATIONS  Describe the methods used to formulate the recommendations and how final decisions were reached. Specify any areas of disagreement and the methods used to resolve them. | Recommendation development process (e.g., steps used in modified Delphi technique, voting procedures that were considered)  Outcomes of the recommendation development process (e.g., extent to which consensus was reached using modified Delphi technique, outcome of voting procedures)  How the process influenced the recommendations (e.g., results of Delphi technique influence final recommendation, alignment with recommendations and the final vote) |
| 11. CONSIDERATION OF BENEFITS AND HARMS  Report the health benefits, side effects, and risks that were considered when formulating the recommendations. | Supporting data and report of benefits  Supporting data and report of harms/side effects/risks  Reporting of the balance/trade-off between benefits and harms/side effects/risks  Recommendations reflect considerations of both benefits and harms/side effects/risks |
| 12. LINK BETWEEN RECOMMENDATIONS AND EVIDENCE  Describe the explicit link between the recommendations and the evidence on which they are based. | How the guideline development group linked and used the evidence to inform recommendations  Link between each recommendation and key evidence (text description and/or reference list)  Link between recommendations and evidence summaries and/or evidence tables in the results section of the guideline |
| 13. EXTERNAL REVIEW  Report the methodology used to conduct the external review. | Purpose and intent of the external review (e.g., to improve quality, gather feedback on draft recommendations, assess applicability and feasibility, disseminate evidence)  Methods taken to undertake the external review (e.g., rating scale, open-ended questions)  Description of the external reviewers (e.g., number, type of reviewers, affiliations)  Outcomes/information gathered from the external review (e.g., summary of key findings)  How the information gathered was used to inform the guideline development process and/or formation of the recommendations (e.g., guideline panel considered results of review in forming final recommendations) |
| 14. UPDATING PROCEDURE  Describe the procedure for updating the guideline. | A statement that the guideline will be updated  Explicit time interval or explicit criteria to guide decisions about when an update will occur  Methodology for the updating procedure |
| DOMAIN 4: CLARITY OF PRESENTATION |  |
| 15. SPECIFIC AND UNAMBIGUOUS RECOMMENDATIONS  Describe which options are appropriate in which situations and in which population groups, as informed by the body of evidence. | A statement of the recommended action  Intent or purpose of the recommended action (e.g., to improve quality of life, to decrease side effects)  Relevant population (e.g., patients, public)  Caveats or qualifying statements, if relevant (e.g., patients or conditions for whom the recommendations would not apply)  If there is uncertainty about the best care option(s), the uncertainty should be stated in the guideline |
| 16. MANAGEMENT OPTIONS  Describe the different options for managing the condition or health issue. | Description of management options  Population or clinical situation most appropriate to each option |
| 17. IDENTIFIABLE KEY RECOMMENDATIONS  Present the key recommendations so that they are easy to identify. | Recommendations in a summarized box, typed in bold, underlined, or presented as flow charts or algorithms  Specific recommendations grouped together in one section |
| DOMAIN 5: APPLICABILITY |  |
| 18. FACILITATORS AND BARRIERS TO APPLICATION  Describe the facilitators and barriers to the guideline’s application. | Types of facilitators and barriers that were considered  Methods by which information regarding the facilitators and barriers to implementing recommendations were sought (e.g., feedback from key stakeholders, pilot testing of guidelines before widespread implementation)  Information/description of the types of facilitators and barriers that emerged from the inquiry (e.g., practitioners have the skills to deliver the recommended care, sufficient equipment is not available to ensure all eligible members of the population receive mammography)  How the information influenced the guideline development process and/or formation of the recommendations |
| 19. IMPLEMENTATION ADVICE/TOOLS  Provide advice and/or tools on how the recommendations can be applied in practice. | Additional materials to support the implementation of the guideline in practice.  For example:   - Guideline summary documents - Links to check lists, algorithms - Links to how-to manuals - Solutions linked to barrier analysis (see Item 18) - Tools to capitalize on guideline facilitators (see Item 18) - Outcome of pilot test and lessons learned |
| 20. RESOURCE IMPLICATIONS  Describe any potential resource implications of applying the recommendations. | Types of cost information that were considered (e.g., economic evaluations, drug acquisition costs)  Methods by which the cost information was sought (e.g., a health economist was part of the guideline development panel, use of health technology assessments for specific drugs, etc.)  Information/description of the cost information that emerged from the inquiry (e.g., specific drug acquisition costs per treatment course)  How the information gathered was used to inform the guideline development process and/or formation of the recommendations |
| 21. MONITORING/ AUDITING CRITERIA  Provide monitoring and/or auditing criteria to measure the application of guideline recommendations. | Criteria to assess guideline implementation or adherence to recommendations  Criteria for assessing impact of implementing the recommendations  Advice on the frequency and interval of measurement  Operational definitions of how the criteria should be measured |
| DOMAIN 6: EDITORIAL INDEPENDENCE |  |
| 22. FUNDING BODY  Report the funding body’s influence on the content of the guideline. | The name of the funding body or source of funding (or explicit statement of no funding)  A statement that the funding body did not influence the content of the guideline |
| 23. COMPETING INTERESTS  Provide an explicit statement that all group members have declared whether they have any competing interests. | Types of competing interests considered  Methods by which potential competing interests were sought  A description of the competing interests  How the competing interests influenced the guideline process and development of recommendations |
| Article: CDC, 2020 |  |
| DOMAIN 1: SCOPE AND PURPOSE | |
| 1. OBJECTIVES  Report the overall objective(s) of the guideline. The expected health benefits from the guideline are to be specific to the clinical problem or health topic. | Health intent(s) (i.e., prevention, screening, diagnosis, treatment, etc.)  Expected benefit(s) or outcome(s)  Target(s) (e.g., patient population, society) |
| 2. QUESTIONS  Report the health question(s) covered by the guideline, particularly for the key recommendations | Intervention(s) or exposure(s)  Comparisons (if appropriate)  Outcome(s)  Health care setting or context |
| 3. POPULATION  Describe the population (i.e., patients, public, etc.) to whom the guideline is meant to apply. | Target population, sex and age  Clinical condition (if relevant)  Severity/stage of disease (if relevant)  Comorbidities (if relevant)  Excluded populations (if relevant) |
| DOMAIN 2: STAKEHOLDER INVOLVEMENT |  |
| 4. GROUP MEMBERSHIP  Report all individuals who were involved in the development process. This may include members of the steering group, the research team involved in selecting and reviewing/rating the evidence and individuals involved in formulating the final recommendations | Name of participant  Discipline/content expertise (e.g., neurosurgeon, methodologist)  Institution (e.g., St. Peter’s hospital)  Geographical location (e.g., Seattle, WA)  A description of the member’s role in the guideline development group |
| 5. TARGET POPULATION PREFERENCES AND VIEWS  Report how the views and preferences of the target population were sought/considered and what the resulting outcomes were. | Statement of type of strategy used to capture patients’/publics’ views and preferences (e.g., participation in the guideline development group, literature review of values and preferences)  Methods by which preferences and views were sought (e.g., evidence from literature, surveys, focus groups)  Outcomes/information gathered on patient/public information  How the information gathered was used to inform the guideline development process and/or formation of the recommendations |
| 6. TARGET USERS  Report the target (or intended) users of the guideline | The intended guideline audience (e.g. specialists, family physicians, patients, clinical or institutional leaders/administrators)  How the guideline may be used by its target audience (e.g., to inform clinical decisions, to inform policy, to inform standards of care) |
| DOMAIN 3: RIGOUR OF DEVELOPMENT |  |
| 7. SEARCH METHODS  Report details of the strategy used to search for evidence. | Named electronic database(s) or evidence source(s) where the search was performed (e.g., MEDLINE, EMBASE, PsychINFO, CINAHL)  Time periods searched (e.g., January 1, 2004 to March 31, 2008)  Search terms used (e.g., text words, indexing terms, subheadings)  Full search strategy included (e.g., possibly located in appendix) |
| 8. EVIDENCE SELECTION CRITERIA  Report the criteria used to select (i.e., include and exclude) the evidence. Provide rationale, where appropriate. | Target population (patient, public, etc.) characteristics  Study design  Comparisons (if relevant)  Outcomes  Language (if relevant)  Context (if relevant) |
| 9. STRENGTHS & LIMITATIONS OF THE EVIDENCE  Describe the strengths and limitations of the evidence. Consider from the perspective of the individual studies and the body of evidence aggregated across all the studies. Tools exist that can facilitate the reporting of this concept. | Study design(s) included in body of evidence  Study methodology limitations (sampling, blinding, allocation concealment, analytical methods)  Appropriateness/relevance of primary and secondary outcomes considered  Consistency of results across studies  Direction of results across studies  Magnitude of benefit versus magnitude of harm  Applicability to practice context |
| 10. FORMULATION OF RECOMMENDATIONS  Describe the methods used to formulate the recommendations and how final decisions were reached. Specify any areas of disagreement and the methods used to resolve them. | Recommendation development process (e.g., steps used in modified Delphi technique, voting procedures that were considered)  Outcomes of the recommendation development process (e.g., extent to which consensus was reached using modified Delphi technique, outcome of voting procedures)  How the process influenced the recommendations (e.g., results of Delphi technique influence final recommendation, alignment with recommendations and the final vote) |
| 11. CONSIDERATION OF BENEFITS AND HARMS  Report the health benefits, side effects, and risks that were considered when formulating the recommendations. | Supporting data and report of benefits  Supporting data and report of harms/side effects/risks  Reporting of the balance/trade-off between benefits and harms/side effects/risks  Recommendations reflect considerations of both benefits and harms/side effects/risks |
| 12. LINK BETWEEN RECOMMENDATIONS AND EVIDENCE  Describe the explicit link between the recommendations and the evidence on which they are based. | How the guideline development group linked and used the evidence to inform recommendations  Link between each recommendation and key evidence (text description and/or reference list)  Link between recommendations and evidence summaries and/or evidence tables in the results section of the guideline |
| 13. EXTERNAL REVIEW  Report the methodology used to conduct the external review. | Purpose and intent of the external review (e.g., to improve quality, gather feedback on draft recommendations, assess applicability and feasibility, disseminate evidence)  Methods taken to undertake the external review (e.g., rating scale, open-ended questions)  Description of the external reviewers (e.g., number, type of reviewers, affiliations)  Outcomes/information gathered from the external review (e.g., summary of key findings)  How the information gathered was used to inform the guideline development process and/or formation of the recommendations (e.g., guideline panel considered results of review in forming final recommendations) |
| 14. UPDATING PROCEDURE  Describe the procedure for updating the guideline. | A statement that the guideline will be updated  Explicit time interval or explicit criteria to guide decisions about when an update will occur  Methodology for the updating procedure |
| DOMAIN 4: CLARITY OF PRESENTATION |  |
| 15. SPECIFIC AND UNAMBIGUOUS RECOMMENDATIONS  Describe which options are appropriate in which situations and in which population groups, as informed by the body of evidence. | A statement of the recommended action  Intent or purpose of the recommended action (e.g., to improve quality of life, to decrease side effects)  Relevant population (e.g., patients, public)  Caveats or qualifying statements, if relevant (e.g., patients or conditions for whom the recommendations would not apply)  If there is uncertainty about the best care option(s), the uncertainty should be stated in the guideline |
| 16. MANAGEMENT OPTIONS  Describe the different options for managing the condition or health issue. | Description of management options  Population or clinical situation most appropriate to each option |
| 17. IDENTIFIABLE KEY RECOMMENDATIONS  Present the key recommendations so that they are easy to identify. | Recommendations in a summarized box, typed in bold, underlined, or presented as flow charts or algorithms  Specific recommendations grouped together in one section |
| DOMAIN 5: APPLICABILITY |  |
| 18. FACILITATORS AND BARRIERS TO APPLICATION  Describe the facilitators and barriers to the guideline’s application. | Types of facilitators and barriers that were considered  Methods by which information regarding the facilitators and barriers to implementing recommendations were sought (e.g., feedback from key stakeholders, pilot testing of guidelines before widespread implementation)  Information/description of the types of facilitators and barriers that emerged from the inquiry (e.g., practitioners have the skills to deliver the recommended care, sufficient equipment is not available to ensure all eligible members of the population receive mammography)  How the information influenced the guideline development process and/or formation of the recommendations |
| 19. IMPLEMENTATION ADVICE/TOOLS  Provide advice and/or tools on how the recommendations can be applied in practice. | Additional materials to support the implementation of the guideline in practice.  For example:   - Guideline summary documents - Links to check lists, algorithms - Links to how-to manuals - Solutions linked to barrier analysis (see Item 18) - Tools to capitalize on guideline facilitators (see Item 18) - Outcome of pilot test and lessons learned |
| 20. RESOURCE IMPLICATIONS  Describe any potential resource implications of applying the recommendations. | Types of cost information that were considered (e.g., economic evaluations, drug acquisition costs)  Methods by which the cost information was sought (e.g., a health economist was part of the guideline development panel, use of health technology assessments for specific drugs, etc.)  Information/description of the cost information that emerged from the inquiry (e.g., specific drug acquisition costs per treatment course)  How the information gathered was used to inform the guideline development process and/or formation of the recommendations |
| 21. MONITORING/ AUDITING CRITERIA  Provide monitoring and/or auditing criteria to measure the application of guideline recommendations. | Criteria to assess guideline implementation or adherence to recommendations  Criteria for assessing impact of implementing the recommendations  Advice on the frequency and interval of measurement  Operational definitions of how the criteria should be measured |
| DOMAIN 6: EDITORIAL INDEPENDENCE |  |
| 22. FUNDING BODY  Report the funding body’s influence on the content of the guideline. | The name of the funding body or source of funding (or explicit statement of no funding)  A statement that the funding body did not influence the content of the guideline |
| 23. COMPETING INTERESTS  Provide an explicit statement that all group members have declared whether they have any competing interests. | Types of competing interests considered  Methods by which potential competing interests were sought  A description of the competing interests  How the competing interests influenced the guideline process and development of recommendations |
| Article: ECRI, 2020a |  |
| DOMAIN 1: SCOPE AND PURPOSE |  |
| 1. OBJECTIVES  Report the overall objective(s) of the guideline. The expected health benefits from the guideline are to be specific to the clinical problem or health topic. | Health intent(s) (i.e., prevention, screening, diagnosis, treatment, etc.)  Expected benefit(s) or outcome(s)  Target(s) (e.g., patient population, society) |
| 2. QUESTIONS  Report the health question(s) covered by the guideline, particularly for the key recommendations | Intervention(s) or exposure(s)  Comparisons (if appropriate)  Outcome(s)  Health care setting or context |
| 3. POPULATION  Describe the population (i.e., patients, public, etc.) to whom the guideline is meant to apply. | Target population, sex and age  Clinical condition (if relevant)  Severity/stage of disease (if relevant)  Comorbidities (if relevant)  Excluded populations (if relevant) |
| DOMAIN 2: STAKEHOLDER INVOLVEMENT |  |
| 4. GROUP MEMBERSHIP  Report all individuals who were involved in the development process. This may include members of the steering group, the research team involved in selecting and reviewing/rating the evidence and individuals involved in formulating the final recommendations | Name of participant  Discipline/content expertise (e.g., neurosurgeon, methodologist)  Institution (e.g., St. Peter’s hospital)  Geographical location (e.g., Seattle, WA)  A description of the member’s role in the guideline development group |
| 5. TARGET POPULATION PREFERENCES AND VIEWS  Report how the views and preferences of the target population were sought/considered and what the resulting outcomes were. | Statement of type of strategy used to capture patients’/publics’ views and preferences (e.g., participation in the guideline development group, literature review of values and preferences)  Methods by which preferences and views were sought (e.g., evidence from literature, surveys, focus groups)  Outcomes/information gathered on patient/public information  How the information gathered was used to inform the guideline development process and/or formation of the recommendations |
| 6. TARGET USERS  Report the target (or intended) users of the guideline | The intended guideline audience (e.g. specialists, family physicians, patients, clinical or institutional leaders/administrators)  How the guideline may be used by its target audience (e.g., to inform clinical decisions, to inform policy, to inform standards of care) |
| DOMAIN 3: RIGOUR OF DEVELOPMENT |  |
| 7. SEARCH METHODS  Report details of the strategy used to search for evidence. | Named electronic database(s) or evidence source(s) where the search was performed (e.g., MEDLINE, EMBASE, PsychINFO, CINAHL)  Time periods searched (e.g., January 1, 2004 to March 31, 2008)  Search terms used (e.g., text words, indexing terms, subheadings)  Full search strategy included (e.g., possibly located in appendix) |
| 8. EVIDENCE SELECTION CRITERIA  Report the criteria used to select (i.e., include and exclude) the evidence. Provide rationale, where appropriate. | Target population (patient, public, etc.) characteristics  Study design  Comparisons (if relevant)  Outcomes  Language (if relevant)  Context (if relevant) |
| 9. STRENGTHS & LIMITATIONS OF THE EVIDENCE  Describe the strengths and limitations of the evidence. Consider from the perspective of the individual studies and the body of evidence aggregated across all the studies. Tools exist that can facilitate the reporting of this concept. | Study design(s) included in body of evidence  Study methodology limitations (sampling, blinding, allocation concealment, analytical methods)  Appropriateness/relevance of primary and secondary outcomes considered  Consistency of results across studies  Direction of results across studies  Magnitude of benefit versus magnitude of harm  Applicability to practice context |
| 10. FORMULATION OF RECOMMENDATIONS  Describe the methods used to formulate the recommendations and how final decisions were reached. Specify any areas of disagreement and the methods used to resolve them. | Recommendation development process (e.g., steps used in modified Delphi technique, voting procedures that were considered)  Outcomes of the recommendation development process (e.g., extent to which consensus was reached using modified Delphi technique, outcome of voting procedures)  How the process influenced the recommendations (e.g., results of Delphi technique influence final recommendation, alignment with recommendations and the final vote) |
| 11. CONSIDERATION OF BENEFITS AND HARMS  Report the health benefits, side effects, and risks that were considered when formulating the recommendations. | Supporting data and report of benefits  Supporting data and report of harms/side effects/risks  Reporting of the balance/trade-off between benefits and harms/side effects/risks  Recommendations reflect considerations of both benefits and harms/side effects/risks |
| 12. LINK BETWEEN RECOMMENDATIONS AND EVIDENCE  Describe the explicit link between the recommendations and the evidence on which they are based. | How the guideline development group linked and used the evidence to inform recommendations  Link between each recommendation and key evidence (text description and/or reference list)  Link between recommendations and evidence summaries and/or evidence tables in the results section of the guideline |
| 13. EXTERNAL REVIEW  Report the methodology used to conduct the external review. | Purpose and intent of the external review (e.g., to improve quality, gather feedback on draft recommendations, assess applicability and feasibility, disseminate evidence)  Methods taken to undertake the external review (e.g., rating scale, open-ended questions)  Description of the external reviewers (e.g., number, type of reviewers, affiliations)  Outcomes/information gathered from the external review (e.g., summary of key findings)  How the information gathered was used to inform the guideline development process and/or formation of the recommendations (e.g., guideline panel considered results of review in forming final recommendations) |
| 14. UPDATING PROCEDURE  Describe the procedure for updating the guideline. | A statement that the guideline will be updated  Explicit time interval or explicit criteria to guide decisions about when an update will occur  Methodology for the updating procedure |
| DOMAIN 4: CLARITY OF PRESENTATION |  |
| 15. SPECIFIC AND UNAMBIGUOUS RECOMMENDATIONS  Describe which options are appropriate in which situations and in which population groups, as informed by the body of evidence. | A statement of the recommended action  Intent or purpose of the recommended action (e.g., to improve quality of life, to decrease side effects)  Relevant population (e.g., patients, public)  Caveats or qualifying statements, if relevant (e.g., patients or conditions for whom the recommendations would not apply)  If there is uncertainty about the best care option(s), the uncertainty should be stated in the guideline |
| 16. MANAGEMENT OPTIONS  Describe the different options for managing the condition or health issue. | Description of management options  Population or clinical situation most appropriate to each option |
| 17. IDENTIFIABLE KEY RECOMMENDATIONS  Present the key recommendations so that they are easy to identify. | Recommendations in a summarized box, typed in bold, underlined, or presented as flow charts or algorithms  Specific recommendations grouped together in one section |
| DOMAIN 5: APPLICABILITY |  |
| 18. FACILITATORS AND BARRIERS TO APPLICATION  Describe the facilitators and barriers to the guideline’s application. | Types of facilitators and barriers that were considered  Methods by which information regarding the facilitators and barriers to implementing recommendations were sought (e.g., feedback from key stakeholders, pilot testing of guidelines before widespread implementation)  Information/description of the types of facilitators and barriers that emerged from the inquiry (e.g., practitioners have the skills to deliver the recommended care, sufficient equipment is not available to ensure all eligible members of the population receive mammography)  How the information influenced the guideline development process and/or formation of the recommendations |
| 19. IMPLEMENTATION ADVICE/TOOLS  Provide advice and/or tools on how the recommendations can be applied in practice. | Additional materials to support the implementation of the guideline in practice.  For example:   - Guideline summary documents - Links to check lists, algorithms - Links to how-to manuals - Solutions linked to barrier analysis (see Item 18) - Tools to capitalize on guideline facilitators (see Item 18) - Outcome of pilot test and lessons learned |
| 20. RESOURCE IMPLICATIONS  Describe any potential resource implications of applying the recommendations. | Types of cost information that were considered (e.g., economic evaluations, drug acquisition costs)  Methods by which the cost information was sought (e.g., a health economist was part of the guideline development panel, use of health technology assessments for specific drugs, etc.)  Information/description of the cost information that emerged from the inquiry (e.g., specific drug acquisition costs per treatment course)  How the information gathered was used to inform the guideline development process and/or formation of the recommendations |
| 21. MONITORING/ AUDITING CRITERIA  Provide monitoring and/or auditing criteria to measure the application of guideline recommendations. | Criteria to assess guideline implementation or adherence to recommendations  Criteria for assessing impact of implementing the recommendations  Advice on the frequency and interval of measurement  Operational definitions of how the criteria should be measured |
| DOMAIN 6: EDITORIAL INDEPENDENCE |  |
| 22. FUNDING BODY  Report the funding body’s influence on the content of the guideline. | The name of the funding body or source of funding (or explicit statement of no funding)  A statement that the funding body did not influence the content of the guideline |
| 23. COMPETING INTERESTS  Provide an explicit statement that all group members have declared whether they have any competing interests. | Types of competing interests considered  Methods by which potential competing interests were sought  A description of the competing interests  How the competing interests influenced the guideline process and development of recommendations |
| Article: ECRI, 2020b |  |
| DOMAIN 1: SCOPE AND PURPOSE |  |
| 1. OBJECTIVES  Report the overall objective(s) of the guideline. The expected health benefits from the guideline are to be specific to the clinical problem or health topic. | Health intent(s) (i.e., prevention, screening, diagnosis, treatment, etc.)  Expected benefit(s) or outcome(s)  Target(s) (e.g., patient population, society) |
| 2. QUESTIONS  Report the health question(s) covered by the guideline, particularly for the key recommendations | Intervention(s) or exposure(s)  Comparisons (if appropriate)  Outcome(s)  Health care setting or context |
| 3. POPULATION  Describe the population (i.e., patients, public, etc.) to whom the guideline is meant to apply. | Target population, sex and age  Clinical condition (if relevant)  Severity/stage of disease (if relevant)  Comorbidities (if relevant)  Excluded populations (if relevant) |
| DOMAIN 2: STAKEHOLDER INVOLVEMENT |  |
| 4. GROUP MEMBERSHIP  Report all individuals who were involved in the development process. This may include members of the steering group, the research team involved in selecting and reviewing/rating the evidence and individuals involved in formulating the final recommendations | Name of participant  Discipline/content expertise (e.g., neurosurgeon, methodologist)  Institution (e.g., St. Peter’s hospital)  Geographical location (e.g., Seattle, WA)  A description of the member’s role in the guideline development group |
| 5. TARGET POPULATION PREFERENCES AND VIEWS  Report how the views and preferences of the target population were sought/considered and what the resulting outcomes were. | Statement of type of strategy used to capture patients’/publics’ views and preferences (e.g., participation in the guideline development group, literature review of values and preferences)  Methods by which preferences and views were sought (e.g., evidence from literature, surveys, focus groups)  Outcomes/information gathered on patient/public information  How the information gathered was used to inform the guideline development process and/or formation of the recommendations |
| 6. TARGET USERS  Report the target (or intended) users of the guideline | The intended guideline audience (e.g. specialists, family physicians, patients, clinical or institutional leaders/administrators)  How the guideline may be used by its target audience (e.g., to inform clinical decisions, to inform policy, to inform standards of care) |
| DOMAIN 3: RIGOUR OF DEVELOPMENT |  |
| 7. SEARCH METHODS  Report details of the strategy used to search for evidence. | Named electronic database(s) or evidence source(s) where the search was performed (e.g., MEDLINE, EMBASE, PsychINFO, CINAHL)  Time periods searched (e.g., January 1, 2004 to March 31, 2008)  Search terms used (e.g., text words, indexing terms, subheadings)  Full search strategy included (e.g., possibly located in appendix) |
| 8. EVIDENCE SELECTION CRITERIA  Report the criteria used to select (i.e., include and exclude) the evidence. Provide rationale, where appropriate. | Target population (patient, public, etc.) characteristics  Study design  Comparisons (if relevant)  Outcomes  Language (if relevant)  Context (if relevant) |
| 9. STRENGTHS & LIMITATIONS OF THE EVIDENCE  Describe the strengths and limitations of the evidence. Consider from the perspective of the individual studies and the body of evidence aggregated across all the studies. Tools exist that can facilitate the reporting of this concept. | Study design(s) included in body of evidence  Study methodology limitations (sampling, blinding, allocation concealment, analytical methods)  Appropriateness/relevance of primary and secondary outcomes considered  Consistency of results across studies  Direction of results across studies  Magnitude of benefit versus magnitude of harm  Applicability to practice context |
| 10. FORMULATION OF RECOMMENDATIONS  Describe the methods used to formulate the recommendations and how final decisions were reached. Specify any areas of disagreement and the methods used to resolve them. | Recommendation development process (e.g., steps used in modified Delphi technique, voting procedures that were considered)  Outcomes of the recommendation development process (e.g., extent to which consensus was reached using modified Delphi technique, outcome of voting procedures)  How the process influenced the recommendations (e.g., results of Delphi technique influence final recommendation, alignment with recommendations and the final vote) |
| 11. CONSIDERATION OF BENEFITS AND HARMS  Report the health benefits, side effects, and risks that were considered when formulating the recommendations. | Supporting data and report of benefits  Supporting data and report of harms/side effects/risks  Reporting of the balance/trade-off between benefits and harms/side effects/risks  Recommendations reflect considerations of both benefits and harms/side effects/risks |
| 12. LINK BETWEEN RECOMMENDATIONS AND EVIDENCE  Describe the explicit link between the recommendations and the evidence on which they are based. | How the guideline development group linked and used the evidence to inform recommendations  Link between each recommendation and key evidence (text description and/or reference list)  Link between recommendations and evidence summaries and/or evidence tables in the results section of the guideline |
| 13. EXTERNAL REVIEW  Report the methodology used to conduct the external review. | Purpose and intent of the external review (e.g., to improve quality, gather feedback on draft recommendations, assess applicability and feasibility, disseminate evidence)  Methods taken to undertake the external review (e.g., rating scale, open-ended questions)  Description of the external reviewers (e.g., number, type of reviewers, affiliations)  Outcomes/information gathered from the external review (e.g., summary of key findings)  How the information gathered was used to inform the guideline development process and/or formation of the recommendations (e.g., guideline panel considered results of review in forming final recommendations) |
| 14. UPDATING PROCEDURE  Describe the procedure for updating the guideline. | A statement that the guideline will be updated  Explicit time interval or explicit criteria to guide decisions about when an update will occur  Methodology for the updating procedure |
| DOMAIN 4: CLARITY OF PRESENTATION |  |
| 15. SPECIFIC AND UNAMBIGUOUS RECOMMENDATIONS  Describe which options are appropriate in which situations and in which population groups, as informed by the body of evidence. | A statement of the recommended action  Intent or purpose of the recommended action (e.g., to improve quality of life, to decrease side effects)  Relevant population (e.g., patients, public)  Caveats or qualifying statements, if relevant (e.g., patients or conditions for whom the recommendations would not apply)  If there is uncertainty about the best care option(s), the uncertainty should be stated in the guideline |
| 16. MANAGEMENT OPTIONS  Describe the different options for managing the condition or health issue. | Description of management options  Population or clinical situation most appropriate to each option |
| 17. IDENTIFIABLE KEY RECOMMENDATIONS  Present the key recommendations so that they are easy to identify. | Recommendations in a summarized box, typed in bold, underlined, or presented as flow charts or algorithms  Specific recommendations grouped together in one section |
| DOMAIN 5: APPLICABILITY |  |
| 18. FACILITATORS AND BARRIERS TO APPLICATION  Describe the facilitators and barriers to the guideline’s application. | Types of facilitators and barriers that were considered  Methods by which information regarding the facilitators and barriers to implementing recommendations were sought (e.g., feedback from key stakeholders, pilot testing of guidelines before widespread implementation)  Information/description of the types of facilitators and barriers that emerged from the inquiry (e.g., practitioners have the skills to deliver the recommended care, sufficient equipment is not available to ensure all eligible members of the population receive mammography)  How the information influenced the guideline development process and/or formation of the recommendations |
| 19. IMPLEMENTATION ADVICE/TOOLS  Provide advice and/or tools on how the recommendations can be applied in practice. | Additional materials to support the implementation of the guideline in practice.  For example:   - Guideline summary documents - Links to check lists, algorithms - Links to how-to manuals - Solutions linked to barrier analysis (see Item 18) - Tools to capitalize on guideline facilitators (see Item 18) - Outcome of pilot test and lessons learned |
| 20. RESOURCE IMPLICATIONS  Describe any potential resource implications of applying the recommendations. | Types of cost information that were considered (e.g., economic evaluations, drug acquisition costs)  Methods by which the cost information was sought (e.g., a health economist was part of the guideline development panel, use of health technology assessments for specific drugs, etc.)  Information/description of the cost information that emerged from the inquiry (e.g., specific drug acquisition costs per treatment course)  How the information gathered was used to inform the guideline development process and/or formation of the recommendations |
| 21. MONITORING/ AUDITING CRITERIA  Provide monitoring and/or auditing criteria to measure the application of guideline recommendations. | Criteria to assess guideline implementation or adherence to recommendations  Criteria for assessing impact of implementing the recommendations  Advice on the frequency and interval of measurement  Operational definitions of how the criteria should be measured |
| DOMAIN 6: EDITORIAL INDEPENDENCE |  |
| 22. FUNDING BODY  Report the funding body’s influence on the content of the guideline. | The name of the funding body or source of funding (or explicit statement of no funding)  A statement that the funding body did not influence the content of the guideline |
| 23. COMPETING INTERESTS  Provide an explicit statement that all group members have declared whether they have any competing interests. | Types of competing interests considered  Methods by which potential competing interests were sought  A description of the competing interests  How the competing interests influenced the guideline process and development of recommendations |
| Article: Geffen, 2020 |  |
| DOMAIN 1: SCOPE AND PURPOSE |  |
| 1. OBJECTIVES  Report the overall objective(s) of the guideline. The expected health benefits from the guideline are to be specific to the clinical problem or health topic. | Health intent(s) (i.e., prevention, screening, diagnosis, treatment, etc.)  Expected benefit(s) or outcome(s)  Target(s) (e.g., patient population, society) |
| 2. QUESTIONS  Report the health question(s) covered by the guideline, particularly for the key recommendations | Intervention(s) or exposure(s)  Comparisons (if appropriate)  Outcome(s)  Health care setting or context |
| 3. POPULATION  Describe the population (i.e., patients, public, etc.) to whom the guideline is meant to apply. | Target population, sex and age  Clinical condition (if relevant)  Severity/stage of disease (if relevant)  Comorbidities (if relevant)  Excluded populations (if relevant) |
| DOMAIN 2: STAKEHOLDER INVOLVEMENT |  |
| 4. GROUP MEMBERSHIP  Report all individuals who were involved in the development process. This may include members of the steering group, the research team involved in selecting and reviewing/rating the evidence and individuals involved in formulating the final recommendations | Name of participant  Discipline/content expertise (e.g., neurosurgeon, methodologist)  Institution (e.g., St. Peter’s hospital)  Geographical location (e.g., Seattle, WA)  A description of the member’s role in the guideline development group |
| 5. TARGET POPULATION PREFERENCES AND VIEWS  Report how the views and preferences of the target population were sought/considered and what the resulting outcomes were. | Statement of type of strategy used to capture patients’/publics’ views and preferences (e.g., participation in the guideline development group, literature review of values and preferences)  Methods by which preferences and views were sought (e.g., evidence from literature, surveys, focus groups)  Outcomes/information gathered on patient/public information  How the information gathered was used to inform the guideline development process and/or formation of the recommendations |
| 6. TARGET USERS  Report the target (or intended) users of the guideline | The intended guideline audience (e.g. specialists, family physicians, patients, clinical or institutional leaders/administrators)  How the guideline may be used by its target audience (e.g., to inform clinical decisions, to inform policy, to inform standards of care) |
| DOMAIN 3: RIGOUR OF DEVELOPMENT |  |
| 7. SEARCH METHODS  Report details of the strategy used to search for evidence. | Named electronic database(s) or evidence source(s) where the search was performed (e.g., MEDLINE, EMBASE, PsychINFO, CINAHL)  Time periods searched (e.g., January 1, 2004 to March 31, 2008)  Search terms used (e.g., text words, indexing terms, subheadings)  Full search strategy included (e.g., possibly located in appendix) |
| 8. EVIDENCE SELECTION CRITERIA  Report the criteria used to select (i.e., include and exclude) the evidence. Provide rationale, where appropriate. | Target population (patient, public, etc.) characteristics  Study design  Comparisons (if relevant)  Outcomes  Language (if relevant)  Context (if relevant) |
| 9. STRENGTHS & LIMITATIONS OF THE EVIDENCE  Describe the strengths and limitations of the evidence. Consider from the perspective of the individual studies and the body of evidence aggregated across all the studies. Tools exist that can facilitate the reporting of this concept. | Study design(s) included in body of evidence  Study methodology limitations (sampling, blinding, allocation concealment, analytical methods)  Appropriateness/relevance of primary and secondary outcomes considered  Consistency of results across studies  Direction of results across studies  Magnitude of benefit versus magnitude of harm  Applicability to practice context |
| 10. FORMULATION OF RECOMMENDATIONS  Describe the methods used to formulate the recommendations and how final decisions were reached. Specify any areas of disagreement and the methods used to resolve them. | Recommendation development process (e.g., steps used in modified Delphi technique, voting procedures that were considered)  Outcomes of the recommendation development process (e.g., extent to which consensus was reached using modified Delphi technique, outcome of voting procedures)  How the process influenced the recommendations (e.g., results of Delphi technique influence final recommendation, alignment with recommendations and the final vote) |
| 11. CONSIDERATION OF BENEFITS AND HARMS  Report the health benefits, side effects, and risks that were considered when formulating the recommendations. | Supporting data and report of benefits  Supporting data and report of harms/side effects/risks  Reporting of the balance/trade-off between benefits and harms/side effects/risks  Recommendations reflect considerations of both benefits and harms/side effects/risks |
| 12. LINK BETWEEN RECOMMENDATIONS AND EVIDENCE  Describe the explicit link between the recommendations and the evidence on which they are based. | How the guideline development group linked and used the evidence to inform recommendations  Link between each recommendation and key evidence (text description and/or reference list)  Link between recommendations and evidence summaries and/or evidence tables in the results section of the guideline |
| 13. EXTERNAL REVIEW  Report the methodology used to conduct the external review. | Purpose and intent of the external review (e.g., to improve quality, gather feedback on draft recommendations, assess applicability and feasibility, disseminate evidence)  Methods taken to undertake the external review (e.g., rating scale, open-ended questions)  Description of the external reviewers (e.g., number, type of reviewers, affiliations)  Outcomes/information gathered from the external review (e.g., summary of key findings)  How the information gathered was used to inform the guideline development process and/or formation of the recommendations (e.g., guideline panel considered results of review in forming final recommendations) |
| 14. UPDATING PROCEDURE  Describe the procedure for updating the guideline. | A statement that the guideline will be updated  Explicit time interval or explicit criteria to guide decisions about when an update will occur  Methodology for the updating procedure |
| DOMAIN 4: CLARITY OF PRESENTATION |  |
| 15. SPECIFIC AND UNAMBIGUOUS RECOMMENDATIONS  Describe which options are appropriate in which situations and in which population groups, as informed by the body of evidence. | A statement of the recommended action  Intent or purpose of the recommended action (e.g., to improve quality of life, to decrease side effects)  Relevant population (e.g., patients, public)  Caveats or qualifying statements, if relevant (e.g., patients or conditions for whom the recommendations would not apply)  If there is uncertainty about the best care option(s), the uncertainty should be stated in the guideline |
| 16. MANAGEMENT OPTIONS  Describe the different options for managing the condition or health issue. | Description of management options  Population or clinical situation most appropriate to each option |
| 17. IDENTIFIABLE KEY RECOMMENDATIONS  Present the key recommendations so that they are easy to identify. | Recommendations in a summarized box, typed in bold, underlined, or presented as flow charts or algorithms  Specific recommendations grouped together in one section |
| DOMAIN 5: APPLICABILITY |  |
| 18. FACILITATORS AND BARRIERS TO APPLICATION  Describe the facilitators and barriers to the guideline’s application. | Types of facilitators and barriers that were considered  Methods by which information regarding the facilitators and barriers to implementing recommendations were sought (e.g., feedback from key stakeholders, pilot testing of guidelines before widespread implementation)  Information/description of the types of facilitators and barriers that emerged from the inquiry (e.g., practitioners have the skills to deliver the recommended care, sufficient equipment is not available to ensure all eligible members of the population receive mammography)  How the information influenced the guideline development process and/or formation of the recommendations |
| 19. IMPLEMENTATION ADVICE/TOOLS  Provide advice and/or tools on how the recommendations can be applied in practice. | Additional materials to support the implementation of the guideline in practice.  For example:   - Guideline summary documents - Links to check lists, algorithms - Links to how-to manuals - Solutions linked to barrier analysis (see Item 18) - Tools to capitalize on guideline facilitators (see Item 18) - Outcome of pilot test and lessons learned |
| 20. RESOURCE IMPLICATIONS  Describe any potential resource implications of applying the recommendations. | Types of cost information that were considered (e.g., economic evaluations, drug acquisition costs)  Methods by which the cost information was sought (e.g., a health economist was part of the guideline development panel, use of health technology assessments for specific drugs, etc.)  Information/description of the cost information that emerged from the inquiry (e.g., specific drug acquisition costs per treatment course)  How the information gathered was used to inform the guideline development process and/or formation of the recommendations |
| 21. MONITORING/ AUDITING CRITERIA  Provide monitoring and/or auditing criteria to measure the application of guideline recommendations. | Criteria to assess guideline implementation or adherence to recommendations  Criteria for assessing impact of implementing the recommendations  Advice on the frequency and interval of measurement  Operational definitions of how the criteria should be measured |
| DOMAIN 6: EDITORIAL INDEPENDENCE |  |
| 22. FUNDING BODY  Report the funding body’s influence on the content of the guideline. | The name of the funding body or source of funding (or explicit statement of no funding)  A statement that the funding body did not influence the content of the guideline |
| 23. COMPETING INTERESTS  Provide an explicit statement that all group members have declared whether they have any competing interests. | Types of competing interests considered  Methods by which potential competing interests were sought  A description of the competing interests  How the competing interests influenced the guideline process and development of recommendations |
| Article: HPS, 2020 |  |
| DOMAIN 1: SCOPE AND PURPOSE |  |
| 1. OBJECTIVES  Report the overall objective(s) of the guideline. The expected health benefits from the guideline are to be specific to the clinical problem or health topic. | Health intent(s) (i.e., prevention, screening, diagnosis, treatment, etc.)  Expected benefit(s) or outcome(s)  Target(s) (e.g., patient population, society) |
| 2. QUESTIONS  Report the health question(s) covered by the guideline, particularly for the key recommendations | Intervention(s) or exposure(s)  Comparisons (if appropriate)  Outcome(s)  Health care setting or context |
| 3. POPULATION  Describe the population (i.e., patients, public, etc.) to whom the guideline is meant to apply. | Target population, sex and age  Clinical condition (if relevant)  Severity/stage of disease (if relevant)  Comorbidities (if relevant)  Excluded populations (if relevant) |
| DOMAIN 2: STAKEHOLDER INVOLVEMENT |  |
| 4. GROUP MEMBERSHIP  Report all individuals who were involved in the development process. This may include members of the steering group, the research team involved in selecting and reviewing/rating the evidence and individuals involved in formulating the final recommendations | Name of participant  Discipline/content expertise (e.g., neurosurgeon, methodologist)  Institution (e.g., St. Peter’s hospital)  Geographical location (e.g., Seattle, WA)  A description of the member’s role in the guideline development group |
| 5. TARGET POPULATION PREFERENCES AND VIEWS  Report how the views and preferences of the target population were sought/considered and what the resulting outcomes were. | Statement of type of strategy used to capture patients’/publics’ views and preferences (e.g., participation in the guideline development group, literature review of values and preferences)  Methods by which preferences and views were sought (e.g., evidence from literature, surveys, focus groups)  Outcomes/information gathered on patient/public information  How the information gathered was used to inform the guideline development process and/or formation of the recommendations |
| 6. TARGET USERS  Report the target (or intended) users of the guideline | The intended guideline audience (e.g. specialists, family physicians, patients, clinical or institutional leaders/administrators)  How the guideline may be used by its target audience (e.g., to inform clinical decisions, to inform policy, to inform standards of care) |
| DOMAIN 3: RIGOUR OF DEVELOPMENT |  |
| 7. SEARCH METHODS  Report details of the strategy used to search for evidence. | Named electronic database(s) or evidence source(s) where the search was performed (e.g., MEDLINE, EMBASE, PsychINFO, CINAHL)  Time periods searched (e.g., January 1, 2004 to March 31, 2008)  Search terms used (e.g., text words, indexing terms, subheadings)  Full search strategy included (e.g., possibly located in appendix) |
| 8. EVIDENCE SELECTION CRITERIA  Report the criteria used to select (i.e., include and exclude) the evidence. Provide rationale, where appropriate. | Target population (patient, public, etc.) characteristics  Study design  Comparisons (if relevant)  Outcomes  Language (if relevant)  Context (if relevant) |
| 9. STRENGTHS & LIMITATIONS OF THE EVIDENCE  Describe the strengths and limitations of the evidence. Consider from the perspective of the individual studies and the body of evidence aggregated across all the studies. Tools exist that can facilitate the reporting of this concept. | Study design(s) included in body of evidence  Study methodology limitations (sampling, blinding, allocation concealment, analytical methods)  Appropriateness/relevance of primary and secondary outcomes considered  Consistency of results across studies  Direction of results across studies  Magnitude of benefit versus magnitude of harm  Applicability to practice context |
| 10. FORMULATION OF RECOMMENDATIONS  Describe the methods used to formulate the recommendations and how final decisions were reached. Specify any areas of disagreement and the methods used to resolve them. | Recommendation development process (e.g., steps used in modified Delphi technique, voting procedures that were considered)  Outcomes of the recommendation development process (e.g., extent to which consensus was reached using modified Delphi technique, outcome of voting procedures)  How the process influenced the recommendations (e.g., results of Delphi technique influence final recommendation, alignment with recommendations and the final vote) |
| 11. CONSIDERATION OF BENEFITS AND HARMS  Report the health benefits, side effects, and risks that were considered when formulating the recommendations. | Supporting data and report of benefits  Supporting data and report of harms/side effects/risks  Reporting of the balance/trade-off between benefits and harms/side effects/risks  Recommendations reflect considerations of both benefits and harms/side effects/risks |
| 12. LINK BETWEEN RECOMMENDATIONS AND EVIDENCE  Describe the explicit link between the recommendations and the evidence on which they are based. | How the guideline development group linked and used the evidence to inform recommendations  Link between each recommendation and key evidence (text description and/or reference list)  Link between recommendations and evidence summaries and/or evidence tables in the results section of the guideline |
| 13. EXTERNAL REVIEW  Report the methodology used to conduct the external review. | Purpose and intent of the external review (e.g., to improve quality, gather feedback on draft recommendations, assess applicability and feasibility, disseminate evidence)  Methods taken to undertake the external review (e.g., rating scale, open-ended questions)  Description of the external reviewers (e.g., number, type of reviewers, affiliations)  Outcomes/information gathered from the external review (e.g., summary of key findings)  How the information gathered was used to inform the guideline development process and/or formation of the recommendations (e.g., guideline panel considered results of review in forming final recommendations) |
| 14. UPDATING PROCEDURE  Describe the procedure for updating the guideline. | A statement that the guideline will be updated  Explicit time interval or explicit criteria to guide decisions about when an update will occur  Methodology for the updating procedure |
| DOMAIN 4: CLARITY OF PRESENTATION |  |
| 15. SPECIFIC AND UNAMBIGUOUS RECOMMENDATIONS  Describe which options are appropriate in which situations and in which population groups, as informed by the body of evidence. | A statement of the recommended action  Intent or purpose of the recommended action (e.g., to improve quality of life, to decrease side effects)  Relevant population (e.g., patients, public)  Caveats or qualifying statements, if relevant (e.g., patients or conditions for whom the recommendations would not apply)  If there is uncertainty about the best care option(s), the uncertainty should be stated in the guideline |
| 16. MANAGEMENT OPTIONS  Describe the different options for managing the condition or health issue. | Description of management options  Population or clinical situation most appropriate to each option |
| 17. IDENTIFIABLE KEY RECOMMENDATIONS  Present the key recommendations so that they are easy to identify. | Recommendations in a summarized box, typed in bold, underlined, or presented as flow charts or algorithms  Specific recommendations grouped together in one section |
| DOMAIN 5: APPLICABILITY |  |
| 18. FACILITATORS AND BARRIERS TO APPLICATION  Describe the facilitators and barriers to the guideline’s application. | Types of facilitators and barriers that were considered  Methods by which information regarding the facilitators and barriers to implementing recommendations were sought (e.g., feedback from key stakeholders, pilot testing of guidelines before widespread implementation)  Information/description of the types of facilitators and barriers that emerged from the inquiry (e.g., practitioners have the skills to deliver the recommended care, sufficient equipment is not available to ensure all eligible members of the population receive mammography)  How the information influenced the guideline development process and/or formation of the recommendations |
| 19. IMPLEMENTATION ADVICE/TOOLS  Provide advice and/or tools on how the recommendations can be applied in practice. | Additional materials to support the implementation of the guideline in practice.  For example:   - Guideline summary documents - Links to check lists, algorithms - Links to how-to manuals - Solutions linked to barrier analysis (see Item 18) - Tools to capitalize on guideline facilitators (see Item 18) - Outcome of pilot test and lessons learned |
| 20. RESOURCE IMPLICATIONS  Describe any potential resource implications of applying the recommendations. | Types of cost information that were considered (e.g., economic evaluations, drug acquisition costs)  Methods by which the cost information was sought (e.g., a health economist was part of the guideline development panel, use of health technology assessments for specific drugs, etc.)  Information/description of the cost information that emerged from the inquiry (e.g., specific drug acquisition costs per treatment course)  How the information gathered was used to inform the guideline development process and/or formation of the recommendations |
| 21. MONITORING/ AUDITING CRITERIA  Provide monitoring and/or auditing criteria to measure the application of guideline recommendations. | Criteria to assess guideline implementation or adherence to recommendations  Criteria for assessing impact of implementing the recommendations  Advice on the frequency and interval of measurement  Operational definitions of how the criteria should be measured |
| DOMAIN 6: EDITORIAL INDEPENDENCE |  |
| 22. FUNDING BODY  Report the funding body’s influence on the content of the guideline. | The name of the funding body or source of funding (or explicit statement of no funding)  A statement that the funding body did not influence the content of the guideline |
| 23. COMPETING INTERESTS  Provide an explicit statement that all group members have declared whether they have any competing interests. | Types of competing interests considered  Methods by which potential competing interests were sought  A description of the competing interests  How the competing interests influenced the guideline process and development of recommendations |
| Article: Lester, 2020 |  |
| DOMAIN 1: SCOPE AND PURPOSE |  |
| 1. OBJECTIVES  Report the overall objective(s) of the guideline. The expected health benefits from the guideline are to be specific to the clinical problem or health topic. | Health intent(s) (i.e., prevention, screening, diagnosis, treatment, etc.)  Expected benefit(s) or outcome(s)  Target(s) (e.g., patient population, society) |
| 2. QUESTIONS  Report the health question(s) covered by the guideline, particularly for the key recommendations | Intervention(s) or exposure(s)  Comparisons (if appropriate)  Outcome(s)  Health care setting or context |
| 3. POPULATION  Describe the population (i.e., patients, public, etc.) to whom the guideline is meant to apply. | Target population, sex and age  Clinical condition (if relevant)  Severity/stage of disease (if relevant)  Comorbidities (if relevant)  Excluded populations (if relevant) |
| DOMAIN 2: STAKEHOLDER INVOLVEMENT |  |
| 4. GROUP MEMBERSHIP  Report all individuals who were involved in the development process. This may include members of the steering group, the research team involved in selecting and reviewing/rating the evidence and individuals involved in formulating the final recommendations | Name of participant  Discipline/content expertise (e.g., neurosurgeon, methodologist)  Institution (e.g., St. Peter’s hospital)  Geographical location (e.g., Seattle, WA)  A description of the member’s role in the guideline development group |
| 5. TARGET POPULATION PREFERENCES AND VIEWS  Report how the views and preferences of the target population were sought/considered and what the resulting outcomes were. | Statement of type of strategy used to capture patients’/publics’ views and preferences (e.g., participation in the guideline development group, literature review of values and preferences)  Methods by which preferences and views were sought (e.g., evidence from literature, surveys, focus groups)  Outcomes/information gathered on patient/public information  How the information gathered was used to inform the guideline development process and/or formation of the recommendations |
| 6. TARGET USERS  Report the target (or intended) users of the guideline | The intended guideline audience (e.g. specialists, family physicians, patients, clinical or institutional leaders/administrators)  How the guideline may be used by its target audience (e.g., to inform clinical decisions, to inform policy, to inform standards of care) |
| DOMAIN 3: RIGOUR OF DEVELOPMENT |  |
| 7. SEARCH METHODS  Report details of the strategy used to search for evidence. | Named electronic database(s) or evidence source(s) where the search was performed (e.g., MEDLINE, EMBASE, PsychINFO, CINAHL)  Time periods searched (e.g., January 1, 2004 to March 31, 2008)  Search terms used (e.g., text words, indexing terms, subheadings)  Full search strategy included (e.g., possibly located in appendix) |
| 8. EVIDENCE SELECTION CRITERIA  Report the criteria used to select (i.e., include and exclude) the evidence. Provide rationale, where appropriate. | Target population (patient, public, etc.) characteristics  Study design  Comparisons (if relevant)  Outcomes  Language (if relevant)  Context (if relevant) |
| 9. STRENGTHS & LIMITATIONS OF THE EVIDENCE  Describe the strengths and limitations of the evidence. Consider from the perspective of the individual studies and the body of evidence aggregated across all the studies. Tools exist that can facilitate the reporting of this concept. | Study design(s) included in body of evidence  Study methodology limitations (sampling, blinding, allocation concealment, analytical methods)  Appropriateness/relevance of primary and secondary outcomes considered  Consistency of results across studies  Direction of results across studies  Magnitude of benefit versus magnitude of harm  Applicability to practice context |
| 10. FORMULATION OF RECOMMENDATIONS  Describe the methods used to formulate the recommendations and how final decisions were reached. Specify any areas of disagreement and the methods used to resolve them. | Recommendation development process (e.g., steps used in modified Delphi technique, voting procedures that were considered)  Outcomes of the recommendation development process (e.g., extent to which consensus was reached using modified Delphi technique, outcome of voting procedures)  How the process influenced the recommendations (e.g., results of Delphi technique influence final recommendation, alignment with recommendations and the final vote) |
| 11. CONSIDERATION OF BENEFITS AND HARMS  Report the health benefits, side effects, and risks that were considered when formulating the recommendations. | Supporting data and report of benefits  Supporting data and report of harms/side effects/risks  Reporting of the balance/trade-off between benefits and harms/side effects/risks  Recommendations reflect considerations of both benefits and harms/side effects/risks |
| 12. LINK BETWEEN RECOMMENDATIONS AND EVIDENCE  Describe the explicit link between the recommendations and the evidence on which they are based. | How the guideline development group linked and used the evidence to inform recommendations  Link between each recommendation and key evidence (text description and/or reference list)  Link between recommendations and evidence summaries and/or evidence tables in the results section of the guideline |
| 13. EXTERNAL REVIEW  Report the methodology used to conduct the external review. | Purpose and intent of the external review (e.g., to improve quality, gather feedback on draft recommendations, assess applicability and feasibility, disseminate evidence)  Methods taken to undertake the external review (e.g., rating scale, open-ended questions)  Description of the external reviewers (e.g., number, type of reviewers, affiliations)  Outcomes/information gathered from the external review (e.g., summary of key findings)  How the information gathered was used to inform the guideline development process and/or formation of the recommendations (e.g., guideline panel considered results of review in forming final recommendations) |
| 14. UPDATING PROCEDURE  Describe the procedure for updating the guideline. | A statement that the guideline will be updated  Explicit time interval or explicit criteria to guide decisions about when an update will occur  Methodology for the updating procedure |
| DOMAIN 4: CLARITY OF PRESENTATION |  |
| 15. SPECIFIC AND UNAMBIGUOUS RECOMMENDATIONS  Describe which options are appropriate in which situations and in which population groups, as informed by the body of evidence. | A statement of the recommended action  Intent or purpose of the recommended action (e.g., to improve quality of life, to decrease side effects)  Relevant population (e.g., patients, public)  Caveats or qualifying statements, if relevant (e.g., patients or conditions for whom the recommendations would not apply)  If there is uncertainty about the best care option(s), the uncertainty should be stated in the guideline |
| 16. MANAGEMENT OPTIONS  Describe the different options for managing the condition or health issue. | Description of management options  Population or clinical situation most appropriate to each option |
| 17. IDENTIFIABLE KEY RECOMMENDATIONS  Present the key recommendations so that they are easy to identify. | Recommendations in a summarized box, typed in bold, underlined, or presented as flow charts or algorithms  Specific recommendations grouped together in one section |
| DOMAIN 5: APPLICABILITY |  |
| 18. FACILITATORS AND BARRIERS TO APPLICATION  Describe the facilitators and barriers to the guideline’s application. | Types of facilitators and barriers that were considered  Methods by which information regarding the facilitators and barriers to implementing recommendations were sought (e.g., feedback from key stakeholders, pilot testing of guidelines before widespread implementation)  Information/description of the types of facilitators and barriers that emerged from the inquiry (e.g., practitioners have the skills to deliver the recommended care, sufficient equipment is not available to ensure all eligible members of the population receive mammography)  How the information influenced the guideline development process and/or formation of the recommendations |
| 19. IMPLEMENTATION ADVICE/TOOLS  Provide advice and/or tools on how the recommendations can be applied in practice. | Additional materials to support the implementation of the guideline in practice.  For example:   - Guideline summary documents - Links to check lists, algorithms - Links to how-to manuals - Solutions linked to barrier analysis (see Item 18) - Tools to capitalize on guideline facilitators (see Item 18) - Outcome of pilot test and lessons learned |
| 20. RESOURCE IMPLICATIONS  Describe any potential resource implications of applying the recommendations. | Types of cost information that were considered (e.g., economic evaluations, drug acquisition costs)  Methods by which the cost information was sought (e.g., a health economist was part of the guideline development panel, use of health technology assessments for specific drugs, etc.)  Information/description of the cost information that emerged from the inquiry (e.g., specific drug acquisition costs per treatment course)  How the information gathered was used to inform the guideline development process and/or formation of the recommendations |
| 21. MONITORING/ AUDITING CRITERIA  Provide monitoring and/or auditing criteria to measure the application of guideline recommendations. | Criteria to assess guideline implementation or adherence to recommendations  Criteria for assessing impact of implementing the recommendations  Advice on the frequency and interval of measurement  Operational definitions of how the criteria should be measured |
| DOMAIN 6: EDITORIAL INDEPENDENCE |  |
| 22. FUNDING BODY  Report the funding body’s influence on the content of the guideline. | The name of the funding body or source of funding (or explicit statement of no funding)  A statement that the funding body did not influence the content of the guideline |
| 23. COMPETING INTERESTS  Provide an explicit statement that all group members have declared whether they have any competing interests. | Types of competing interests considered  Methods by which potential competing interests were sought  A description of the competing interests  How the competing interests influenced the guideline process and development of recommendations |
| Article: MOH, 2020 |  |
| DOMAIN 1: SCOPE AND PURPOSE |  |
| 1. OBJECTIVES  Report the overall objective(s) of the guideline. The expected health benefits from the guideline are to be specific to the clinical problem or health topic. | Health intent(s) (i.e., prevention, screening, diagnosis, treatment, etc.)  Expected benefit(s) or outcome(s)  Target(s) (e.g., patient population, society) |
| 2. QUESTIONS  Report the health question(s) covered by the guideline, particularly for the key recommendations | Intervention(s) or exposure(s)  Comparisons (if appropriate)  Outcome(s)  Health care setting or context |
| 3. POPULATION  Describe the population (i.e., patients, public, etc.) to whom the guideline is meant to apply. | Target population, sex and age  Clinical condition (if relevant)  Severity/stage of disease (if relevant)  Comorbidities (if relevant)  Excluded populations (if relevant) |
| DOMAIN 2: STAKEHOLDER INVOLVEMENT |  |
| 4. GROUP MEMBERSHIP  Report all individuals who were involved in the development process. This may include members of the steering group, the research team involved in selecting and reviewing/rating the evidence and individuals involved in formulating the final recommendations | Name of participant  Discipline/content expertise (e.g., neurosurgeon, methodologist)  Institution (e.g., St. Peter’s hospital)  Geographical location (e.g., Seattle, WA)  A description of the member’s role in the guideline development group |
| 5. TARGET POPULATION PREFERENCES AND VIEWS  Report how the views and preferences of the target population were sought/considered and what the resulting outcomes were. | Statement of type of strategy used to capture patients’/publics’ views and preferences (e.g., participation in the guideline development group, literature review of values and preferences)  Methods by which preferences and views were sought (e.g., evidence from literature, surveys, focus groups)  Outcomes/information gathered on patient/public information  How the information gathered was used to inform the guideline development process and/or formation of the recommendations |
| 6. TARGET USERS  Report the target (or intended) users of the guideline | The intended guideline audience (e.g. specialists, family physicians, patients, clinical or institutional leaders/administrators)  How the guideline may be used by its target audience (e.g., to inform clinical decisions, to inform policy, to inform standards of care) |
| DOMAIN 3: RIGOUR OF DEVELOPMENT |  |
| 7. SEARCH METHODS  Report details of the strategy used to search for evidence. | Named electronic database(s) or evidence source(s) where the search was performed (e.g., MEDLINE, EMBASE, PsychINFO, CINAHL)  Time periods searched (e.g., January 1, 2004 to March 31, 2008)  Search terms used (e.g., text words, indexing terms, subheadings)  Full search strategy included (e.g., possibly located in appendix) |
| 8. EVIDENCE SELECTION CRITERIA  Report the criteria used to select (i.e., include and exclude) the evidence. Provide rationale, where appropriate. | Target population (patient, public, etc.) characteristics  Study design  Comparisons (if relevant)  Outcomes  Language (if relevant)  Context (if relevant) |
| 9. STRENGTHS & LIMITATIONS OF THE EVIDENCE  Describe the strengths and limitations of the evidence. Consider from the perspective of the individual studies and the body of evidence aggregated across all the studies. Tools exist that can facilitate the reporting of this concept. | Study design(s) included in body of evidence  Study methodology limitations (sampling, blinding, allocation concealment, analytical methods)  Appropriateness/relevance of primary and secondary outcomes considered  Consistency of results across studies  Direction of results across studies  Magnitude of benefit versus magnitude of harm  Applicability to practice context |
| 10. FORMULATION OF RECOMMENDATIONS  Describe the methods used to formulate the recommendations and how final decisions were reached. Specify any areas of disagreement and the methods used to resolve them. | Recommendation development process (e.g., steps used in modified Delphi technique, voting procedures that were considered)  Outcomes of the recommendation development process (e.g., extent to which consensus was reached using modified Delphi technique, outcome of voting procedures)  How the process influenced the recommendations (e.g., results of Delphi technique influence final recommendation, alignment with recommendations and the final vote) |
| 11. CONSIDERATION OF BENEFITS AND HARMS  Report the health benefits, side effects, and risks that were considered when formulating the recommendations. | Supporting data and report of benefits  Supporting data and report of harms/side effects/risks  Reporting of the balance/trade-off between benefits and harms/side effects/risks  Recommendations reflect considerations of both benefits and harms/side effects/risks |
| 12. LINK BETWEEN RECOMMENDATIONS AND EVIDENCE  Describe the explicit link between the recommendations and the evidence on which they are based. | How the guideline development group linked and used the evidence to inform recommendations  Link between each recommendation and key evidence (text description and/or reference list)  Link between recommendations and evidence summaries and/or evidence tables in the results section of the guideline |
| 13. EXTERNAL REVIEW  Report the methodology used to conduct the external review. | Purpose and intent of the external review (e.g., to improve quality, gather feedback on draft recommendations, assess applicability and feasibility, disseminate evidence)  Methods taken to undertake the external review (e.g., rating scale, open-ended questions)  Description of the external reviewers (e.g., number, type of reviewers, affiliations)  Outcomes/information gathered from the external review (e.g., summary of key findings)  How the information gathered was used to inform the guideline development process and/or formation of the recommendations (e.g., guideline panel considered results of review in forming final recommendations) |
| 14. UPDATING PROCEDURE  Describe the procedure for updating the guideline. | A statement that the guideline will be updated  Explicit time interval or explicit criteria to guide decisions about when an update will occur  Methodology for the updating procedure |
| DOMAIN 4: CLARITY OF PRESENTATION |  |
| 15. SPECIFIC AND UNAMBIGUOUS RECOMMENDATIONS  Describe which options are appropriate in which situations and in which population groups, as informed by the body of evidence. | A statement of the recommended action  Intent or purpose of the recommended action (e.g., to improve quality of life, to decrease side effects)  Relevant population (e.g., patients, public)  Caveats or qualifying statements, if relevant (e.g., patients or conditions for whom the recommendations would not apply)  If there is uncertainty about the best care option(s), the uncertainty should be stated in the guideline |
| 16. MANAGEMENT OPTIONS  Describe the different options for managing the condition or health issue. | Description of management options  Population or clinical situation most appropriate to each option |
| 17. IDENTIFIABLE KEY RECOMMENDATIONS  Present the key recommendations so that they are easy to identify. | Recommendations in a summarized box, typed in bold, underlined, or presented as flow charts or algorithms  Specific recommendations grouped together in one section |
| DOMAIN 5: APPLICABILITY |  |
| 18. FACILITATORS AND BARRIERS TO APPLICATION  Describe the facilitators and barriers to the guideline’s application. | Types of facilitators and barriers that were considered  Methods by which information regarding the facilitators and barriers to implementing recommendations were sought (e.g., feedback from key stakeholders, pilot testing of guidelines before widespread implementation)  Information/description of the types of facilitators and barriers that emerged from the inquiry (e.g., practitioners have the skills to deliver the recommended care, sufficient equipment is not available to ensure all eligible members of the population receive mammography)  How the information influenced the guideline development process and/or formation of the recommendations |
| 19. IMPLEMENTATION ADVICE/TOOLS  Provide advice and/or tools on how the recommendations can be applied in practice. | Additional materials to support the implementation of the guideline in practice.  For example:   - Guideline summary documents - Links to check lists, algorithms - Links to how-to manuals - Solutions linked to barrier analysis (see Item 18) - Tools to capitalize on guideline facilitators (see Item 18) - Outcome of pilot test and lessons learned |
| 20. RESOURCE IMPLICATIONS  Describe any potential resource implications of applying the recommendations. | Types of cost information that were considered (e.g., economic evaluations, drug acquisition costs)  Methods by which the cost information was sought (e.g., a health economist was part of the guideline development panel, use of health technology assessments for specific drugs, etc.)  Information/description of the cost information that emerged from the inquiry (e.g., specific drug acquisition costs per treatment course)  How the information gathered was used to inform the guideline development process and/or formation of the recommendations |
| 21. MONITORING/ AUDITING CRITERIA  Provide monitoring and/or auditing criteria to measure the application of guideline recommendations. | Criteria to assess guideline implementation or adherence to recommendations  Criteria for assessing impact of implementing the recommendations  Advice on the frequency and interval of measurement  Operational definitions of how the criteria should be measured |
| DOMAIN 6: EDITORIAL INDEPENDENCE |  |
| 22. FUNDING BODY  Report the funding body’s influence on the content of the guideline. | The name of the funding body or source of funding (or explicit statement of no funding)  A statement that the funding body did not influence the content of the guideline |
| 23. COMPETING INTERESTS  Provide an explicit statement that all group members have declared whether they have any competing interests. | Types of competing interests considered  Methods by which potential competing interests were sought  A description of the competing interests  How the competing interests influenced the guideline process and development of recommendations |
| Article: WHO, 2020 |  |
| DOMAIN 1: SCOPE AND PURPOSE |  |
| 1. OBJECTIVES  Report the overall objective(s) of the guideline. The expected health benefits from the guideline are to be specific to the clinical problem or health topic. | Health intent(s) (i.e., prevention, screening, diagnosis, treatment, etc.)  Expected benefit(s) or outcome(s)  Target(s) (e.g., patient population, society) |
| 2. QUESTIONS  Report the health question(s) covered by the guideline, particularly for the key recommendations | Intervention(s) or exposure(s)  Comparisons (if appropriate)  Outcome(s)  Health care setting or context |
| 3. POPULATION  Describe the population (i.e., patients, public, etc.) to whom the guideline is meant to apply. | Target population, sex and age  Clinical condition (if relevant)  Severity/stage of disease (if relevant)  Comorbidities (if relevant)  Excluded populations (if relevant) |
| DOMAIN 2: STAKEHOLDER INVOLVEMENT |  |
| 4. GROUP MEMBERSHIP  Report all individuals who were involved in the development process. This may include members of the steering group, the research team involved in selecting and reviewing/rating the evidence and individuals involved in formulating the final recommendations | Name of participant  Discipline/content expertise (e.g., neurosurgeon, methodologist)  Institution (e.g., St. Peter’s hospital)  Geographical location (e.g., Seattle, WA)  A description of the member’s role in the guideline development group |
| 5. TARGET POPULATION PREFERENCES AND VIEWS  Report how the views and preferences of the target population were sought/considered and what the resulting outcomes were. | Statement of type of strategy used to capture patients’/publics’ views and preferences (e.g., participation in the guideline development group, literature review of values and preferences)  Methods by which preferences and views were sought (e.g., evidence from literature, surveys, focus groups)  Outcomes/information gathered on patient/public information  How the information gathered was used to inform the guideline development process and/or formation of the recommendations |
| 6. TARGET USERS  Report the target (or intended) users of the guideline | The intended guideline audience (e.g. specialists, family physicians, patients, clinical or institutional leaders/administrators)  How the guideline may be used by its target audience (e.g., to inform clinical decisions, to inform policy, to inform standards of care) |
| DOMAIN 3: RIGOUR OF DEVELOPMENT |  |
| 7. SEARCH METHODS  Report details of the strategy used to search for evidence. | Named electronic database(s) or evidence source(s) where the search was performed (e.g., MEDLINE, EMBASE, PsychINFO, CINAHL)  Time periods searched (e.g., January 1, 2004 to March 31, 2008)  Search terms used (e.g., text words, indexing terms, subheadings)  Full search strategy included (e.g., possibly located in appendix) |
| 8. EVIDENCE SELECTION CRITERIA  Report the criteria used to select (i.e., include and exclude) the evidence. Provide rationale, where appropriate. | Target population (patient, public, etc.) characteristics  Study design  Comparisons (if relevant)  Outcomes  Language (if relevant)  Context (if relevant) |
| 9. STRENGTHS & LIMITATIONS OF THE EVIDENCE  Describe the strengths and limitations of the evidence. Consider from the perspective of the individual studies and the body of evidence aggregated across all the studies. Tools exist that can facilitate the reporting of this concept. | Study design(s) included in body of evidence  Study methodology limitations (sampling, blinding, allocation concealment, analytical methods)  Appropriateness/relevance of primary and secondary outcomes considered  Consistency of results across studies  Direction of results across studies  Magnitude of benefit versus magnitude of harm  Applicability to practice context |
| 10. FORMULATION OF RECOMMENDATIONS  Describe the methods used to formulate the recommendations and how final decisions were reached. Specify any areas of disagreement and the methods used to resolve them. | Recommendation development process (e.g., steps used in modified Delphi technique, voting procedures that were considered)  Outcomes of the recommendation development process (e.g., extent to which consensus was reached using modified Delphi technique, outcome of voting procedures)  How the process influenced the recommendations (e.g., results of Delphi technique influence final recommendation, alignment with recommendations and the final vote) |
| 11. CONSIDERATION OF BENEFITS AND HARMS  Report the health benefits, side effects, and risks that were considered when formulating the recommendations. | Supporting data and report of benefits  Supporting data and report of harms/side effects/risks  Reporting of the balance/trade-off between benefits and harms/side effects/risks  Recommendations reflect considerations of both benefits and harms/side effects/risks |
| 12. LINK BETWEEN RECOMMENDATIONS AND EVIDENCE  Describe the explicit link between the recommendations and the evidence on which they are based. | How the guideline development group linked and used the evidence to inform recommendations  Link between each recommendation and key evidence (text description and/or reference list)  Link between recommendations and evidence summaries and/or evidence tables in the results section of the guideline |
| 13. EXTERNAL REVIEW  Report the methodology used to conduct the external review. | Purpose and intent of the external review (e.g., to improve quality, gather feedback on draft recommendations, assess applicability and feasibility, disseminate evidence)  Methods taken to undertake the external review (e.g., rating scale, open-ended questions)  Description of the external reviewers (e.g., number, type of reviewers, affiliations)  Outcomes/information gathered from the external review (e.g., summary of key findings)  How the information gathered was used to inform the guideline development process and/or formation of the recommendations (e.g., guideline panel considered results of review in forming final recommendations) |
| 14. UPDATING PROCEDURE  Describe the procedure for updating the guideline. | A statement that the guideline will be updated  Explicit time interval or explicit criteria to guide decisions about when an update will occur  Methodology for the updating procedure |
| DOMAIN 4: CLARITY OF PRESENTATION |  |
| 15. SPECIFIC AND UNAMBIGUOUS RECOMMENDATIONS  Describe which options are appropriate in which situations and in which population groups, as informed by the body of evidence. | A statement of the recommended action  Intent or purpose of the recommended action (e.g., to improve quality of life, to decrease side effects)  Relevant population (e.g., patients, public)  Caveats or qualifying statements, if relevant (e.g., patients or conditions for whom the recommendations would not apply)  If there is uncertainty about the best care option(s), the uncertainty should be stated in the guideline |
| 16. MANAGEMENT OPTIONS  Describe the different options for managing the condition or health issue. | Description of management options  Population or clinical situation most appropriate to each option |
| 17. IDENTIFIABLE KEY RECOMMENDATIONS  Present the key recommendations so that they are easy to identify. | Recommendations in a summarized box, typed in bold, underlined, or presented as flow charts or algorithms  Specific recommendations grouped together in one section |
| DOMAIN 5: APPLICABILITY |  |
| 18. FACILITATORS AND BARRIERS TO APPLICATION  Describe the facilitators and barriers to the guideline’s application. | Types of facilitators and barriers that were considered  Methods by which information regarding the facilitators and barriers to implementing recommendations were sought (e.g., feedback from key stakeholders, pilot testing of guidelines before widespread implementation)  Information/description of the types of facilitators and barriers that emerged from the inquiry (e.g., practitioners have the skills to deliver the recommended care, sufficient equipment is not available to ensure all eligible members of the population receive mammography)  How the information influenced the guideline development process and/or formation of the recommendations |
| 19. IMPLEMENTATION ADVICE/TOOLS  Provide advice and/or tools on how the recommendations can be applied in practice. | Additional materials to support the implementation of the guideline in practice.  For example:   - Guideline summary documents - Links to check lists, algorithms - Links to how-to manuals - Solutions linked to barrier analysis (see Item 18) - Tools to capitalize on guideline facilitators (see Item 18) - Outcome of pilot test and lessons learned |
| 20. RESOURCE IMPLICATIONS  Describe any potential resource implications of applying the recommendations. | Types of cost information that were considered (e.g., economic evaluations, drug acquisition costs)  Methods by which the cost information was sought (e.g., a health economist was part of the guideline development panel, use of health technology assessments for specific drugs, etc.)  Information/description of the cost information that emerged from the inquiry (e.g., specific drug acquisition costs per treatment course)  How the information gathered was used to inform the guideline development process and/or formation of the recommendations |
| 21. MONITORING/ AUDITING CRITERIA  Provide monitoring and/or auditing criteria to measure the application of guideline recommendations. | Criteria to assess guideline implementation or adherence to recommendations  Criteria for assessing impact of implementing the recommendations  Advice on the frequency and interval of measurement  Operational definitions of how the criteria should be measured |
| DOMAIN 6: EDITORIAL INDEPENDENCE |  |
| 22. FUNDING BODY  Report the funding body’s influence on the content of the guideline. | The name of the funding body or source of funding (or explicit statement of no funding)  A statement that the funding body did not influence the content of the guideline |
| 23. COMPETING INTERESTS  Provide an explicit statement that all group members have declared whether they have any competing interests. | Types of competing interests considered  Methods by which potential competing interests were sought  A description of the competing interests  How the competing interests influenced the guideline process and development of recommendations |

# APPENDIX 6 – Clinical Practice Guideline Results

| AGS, 2020; Country: USA, Sponsor: American Geriatrics Society  Scope: recommendations to guide federal, state, and local governments when making decisions about how best to care for patients with COVID-19 in nursing homes (NHs) and other long-term care facilities (LTCFs) |
| --- |
| Increase production and distribution of:   - PPE: This includes the masks, face shields, gowns, and gloves that all frontline healthcare professionals and direct care workers need in order to protect themselves against becoming infected. PPE protects health workers’ own safety, which is key to ensuring we have access to the healthcare workforce we need during this pandemic. - Testing kits and related laboratory supplies: Supplies for diagnostic and serologic testing are integral to protecting the health and safety of all Americans during a pandemic - Supplies for symptom management and end-of-life care: The federal government should proactively monitor the available supply of medications (including opioids) and equipment commonly used in symptom management and at the end of life, particularly for people who develop the distressful and uncomfortable symptoms of respiratory failure. If shortages are imminent, the President should fully exercise his authorities under the Defense Production Act to prevent a gap in the supply of the medicines and equipment critical to symptom management, especially at the end of life.   Supply Chain   - coordinate the sharing of scarce resources within and across states, deliver new resources to states and communities, and help to prioritize, NHs, LTCFs, other congregate living settings (e.g., assisted living), and home health care agencies (e.g., Visiting Nurse Association) for the tools and resources they need. - Individuals who test positive for COVID-19 should not be discharged to a mainstream NH unless the facility can safely and effectively isolate the patient from other residents and has adequate infection control protocols and PPE for staff and residents. Such transfers should be in accordance with current CDC guidance.   Public health planning   - coordination with several important stakeholders such as Geriatrics health professionals, NH leadership teams (e.g., administrators, medical directors, and directors of nursing), Hospice and palliative care experts, and Local collaborations - Hospital discharge also plays an important role in COVID-19 planning. As recommended by the CDC, the first and best option is to discharge to home in isolation with any needed home care. This will involve ensuring that enough home healthcare resources are available to patients who have remaining health needs. It also will involve the use of telemedicine for clinicians to monitor patients discharged to home. Given that this option will likely only be feasible for a small number of patients, the federal government and states should build capacity to care for patients with COVID-19 post hospital-discharge. This includes supporting NHs to readmit their own residents to isolation units or rooms, if available; identifying safe locations for those with wandering behaviors and highly complex care needs; and identifying housing for patients who are not stable enough for discharge to home but who still need support and close monitoring. States should explore “hospital-at-home” models of care, which can provide hospital-level care in the home environment and which should be paid for at parity with institutional hospital care to encourage further adoption - Data also is important to our COVID-19 response. Modelling of hotspots, supply of beds, and PPE must include NHs   Workforce considerations   - Paid leave and assistance for frontline healthcare workers - NHs should implement policies and procedures for screening staff aligned with guidance from the CDC and updated regularly to account for situational change. Infection among staff may be a source of exposure for post-acute patients and long-term residents in NHs. Quarantine rules must be carefully considered so as not to quarantine staff unnecessarily or for too long a period, which could decimate the NH workforce. - All NH staff caring for residents who test positive for COVID-19 should be trained in infection control, the use of PPE, and recognition of COVID-19 symptoms. They also should receive any other training in accordance with federal, state, or local guidance. Resources—including rapidly developed online training tools—should be provided to support innovative training and mentoring for healthcare professionals and workers who are being quickly mobilized into new settings of care - State and local governments should include nursing homes in their emergency personnel distribution deployment considerations. This will ensure adequate and safe staffing ratios for all disciplines providing care to NH residents - CMS should increase payment to NHs caring for residents with COVID-19, so that payment is commensurate with the added costs of enhancing staffing skills, the need for quarantine, and quantities of PPE and other supplies to care for this complex and vulnerable population appropriately |
| CDC, 2020; Country: USA, Sponsor: Centres for Disease Control  Scope: Preventing COVID-19 in nursing homes |
| Nursing homes must act now to implement ALL COVID-19 preparedness recommendations, even before cases are identified in their community  Address asymptomatic and pre-symptomatic transmission, implement source control for everyone entering a healthcare facility (e.g., healthcare personnel, patients, visitors), regardless of symptoms.  Cloth face coverings are not considered personal protective equipment (PPE) because their capability to protect healthcare personnel (HCP) is unknown. Facemasks, if available, should be reserved for HCP.  For visitors and residents, a cloth face covering may be appropriate. If a visitor or resident arrives to the facility without a cloth face covering, a facemask may be used for source control if supplies are available.  Dedicate an area of the facility to care for residents with suspected or confirmed COVID-19; consider creating a staffing plan for that specific location  Educate Residents, Healthcare Personnel, and Visitors about COVID-19, Current Precautions Being Taken in the Facility, and Actions They Can Take to Protect Themselves   - Provide information about COVID-19 (including information about signs and symptoms) and strategies for managing stress and anxiety. - Review CDC’s Interim Infection Prevention and Control Recommendations for Patients with Confirmed Coronavirus Disease 2019 (COVID-19) or Persons Under Investigation for COVID-19 in Healthcare Settings - Educate and train HCP   - Reinforce sick leave policies; remind HCP not to report to work when ill   - Educate them about new policies for source control while in the facility.   - Reinforce adherence to standard infection prevention and control measures including hand hygiene and selection and use of personal protective equipment (PPE). Have HCP demonstrate competency with putting on and removing PPE and monitor adherence by observing resident care activities - Educate both facility-based and consultant personnel (e.g., wound care, podiatry, barber) and volunteers who provide care or services in the facility. Inclusion of consultants is important, since they commonly provide care in multiple facilities and can be exposed to or serve as a source of pathogen transmission - Educate residents and families including, information about COVID-19; actions the facility is taking to protect them and/or their loved ones, including visitor restrictions; and actions they can take to protect themselves in the facility, emphasizing the importance of social distancing, hand hygiene, respiratory hygiene and cough etiquette, and wearing a cloth face covering. - Have a plan and mechanism to regularly communicate with residents, family members and HCP, including if cases of COVID-19 are identified among residents or HCP.   Evaluate and Manage Healthcare Personnel with Symptoms Consistent with COVID-19   - Facilities should implement sick leave policies that are non-punitive, flexible and consistent with public health policies that allow ill HCP to stay home. - Create or review an inventory of all volunteers and personnel who provide care in the facility. Use that inventory to determine which personnel are non-essential and whose services can be delayed. - Review current resident services and restrict non-essential healthcare personnel, such as elective consultations, and volunteers from entering the building.   - Consider implementing telehealth to offer remote access to care activities - As part of source control efforts, HCP should wear a facemask or cloth face covering at all times while they are in the healthcare facility. When available, facemasks are generally preferred over cloth face coverings for HCP as facemasks offer both source control and protection for the wearer against exposure to splashes and sprays of infectious material from others. If there are shortages of facemasks, facemasks should be prioritized for HCP and then for residents with symptoms of COVID-19 (as supply allows). Guidance on extended use and reuse of facemasks is available. Cloth face coverings should NOT be worn instead of a respirator or facemask if more than source control is required.   - All HCP should be reminded to practice social distancing when in break rooms or common areas. - As part of routine practice, HCP (including consultant personnel and ancillary staff such as environmental and dietary services) should be asked to regularly monitor themselves for fever and symptoms of COVID-19.   - HCP should be reminded to stay home when they are ill.   - If HCP develop fever (T≥100.0^o^ F) or symptoms of COVID-19 while at work they should keep their facemask on, inform their supervisor, and leave the workplace.   - HCP with suspected COVID-19 should be prioritized for testing. - Screen all HCP at the beginning of their shift for fever and symptoms of COVID-19   - Actively take their temperature and document absence of shortness of breath, new or change in cough, sore throat, and muscle aches. If they are ill, have them keep their cloth face covering or facemask on and leave the workplace. Fever is either measured temperature >100 F or subjective fever.   - HCP who work in multiple locations may pose higher risk and should be encouraged to tell facilities if they have had exposure to other facilities with recognized COVID-19 cases. - Facilities should develop (or review existing) plans to mitigate staffing shortages from illness or absenteeism.   - CDC has created guidance to assist facilities with mitigating staffing shortages.   - For guidance on when HCP with suspected or confirmed COVID-19 may return to work refer to Criteria for Return to Work for Healthcare Personnel with Confirmed or Suspected COVID-19 (Interim Guidance)   Enforce Policies and Procedures for Visitors   - Because of the ease of spread in a long-term care setting and the severity of illness that occurs in residents with COVID-19, facilities should immediately restrict all visitation to their facilities except for certain compassionate care reasons, such as end-of-life situations.   - Send letters or emails to families advising them that no visitors will be allowed in the facility except for certain compassionate care situations, such as end of life situations.   - Use of alternative methods for visitation (e.g., video conferencing) should be facilitated by the facility.   - Post signs at the entrances to the facility advising that no visitors may enter the facility.   - Decisions about visitation for compassionate care situations should be made on a case-by-case basis, which should include careful screening of the visitor for fever or symptoms consistent with COVID-19. Those with symptoms should not be permitted to enter the facility. Any visitors that are permitted must wear a cloth face covering while in the building and restrict their visit to the resident’s room or other location designated by the facility. They should also be reminded to frequently perform hand hygiene.   - Ask visitors to inform the facility if they develop fever or symptoms consistent with COVID-19 within 14 days of visiting the facility.   Provide Supplies Necessary to Adhere to Recommended Infection Prevention and Control Practices   - Hand Hygiene Supplies:   - Put alcohol-based hand sanitizer with 60-95% alcohol in every resident room (ideally both inside and outside of the room) and other resident care and common areas (e.g., outside dining hall, in therapy gym).   - Make sure that sinks are well-stocked with soap and paper towels for handwashing. - Respiratory Hygiene and Cough Etiquette:   - Tissues and trash cans are available in common areas and resident rooms for respiratory hygiene and cough etiquette and source control. - Personal Protective Equipment (PPE):   - Assess current PPE supply. Identify health department or healthcare coalition contacts for getting assistance during PPE shortages. Monitor daily PPE use to identify when supplies will run low; use the PPE burn rate calculator or other tools   - Implement strategies to optimize current PPE supply even before shortages occur Bundling resident care and treatment activities to minimize entries into resident room (e.g., having clinical staff clean and disinfect high-touch surfaces when in the room)   - Extended use of respirators, facemasks, and eye protection, which refers to the practice of wearing the same respirator or facemask and eye protection for the care of more than one resident (e.g., for an entire shift). Extreme care must be taken to avoid touching the respirator, facemask or eye protection. If this must occur, HCP should perform hand hygiene immediately before and after contact to prevent contaminating themselves or others.   - Prioritizing gowns for activities where splashes and sprays are anticipated (including aerosol generating procedures) and high-contact resident care activities that provide opportunities for transfer of pathogens to hands and clothing of HCP.   - Developing a process for decontamination and reuse of PPE such as face shields and goggles - Make necessary PPE available in areas where resident care is provided.   - Consider designating staff responsible for stewarding those supplies and monitoring and providing just-in-time feedback promoting appropriate use by staff.   - Facilities should have supplies of facemasks, respirators (if available and the facility has a respiratory protection program with trained, medically cleared, and fit tested HCP), gowns, gloves, and eye protection (i.e., face shield or goggles). - Position a trash can near the exit inside the resident room to make it easy for staff to discard PPE, prior to exiting the room, or before providing care for another resident in the same room. - Consider implementing a respiratory protection program that is compliant with the OSHA respiratory protection standard for employees if not already in place. The program should include medical evaluations, training, and fit testing. - Environmental Cleaning and Disinfection:   - Develop a schedule for regular cleaning and disinfection of shared equipment, frequently touched surfaces in resident rooms and common areas;   - Ensure EPA-registered, hospital-grade disinfectants are available to allow for frequent cleaning of high-touch surfaces and shared resident care equipment.   - Refer to the EPA website for EPA-registered disinfectants that have qualified under EPA’s emerging viral pathogens program for use against SARS-CoV-2.   Dedicate Space in the Facility to Monitor and Care for Residents with COVID-19   - Dedicate space in the facility to care for residents with confirmed COVID-19. This could be a dedicated floor, unit, or wing in the facility or a group of rooms at the end of the unit that will be used to cohort residents with COVID-19. Assign dedicated HCP to work only in this area of the facility. - Have a plan for how residents in the facility who develop COVID-19 will be handled (e.g., transfer to single room, prioritize for testing, transfer to COVID-19 unit if positive).   - Closely monitor roommates and other residents who may have been exposed to an individual with COVID-19 and, if possible, avoid placing unexposed residents into a shared space with them. - Create a plan for managing new admissions and readmissions whose COVID-19 status is unknown. Options may include placing the resident in a single-person room or in a separate observation area so the resident can be monitored for evidence of COVID-19. Residents could be transferred out of the observation area to the main facility if they remain afebrile and without symptoms for 14 days after their exposure (or admission). Testing at the end of this period could be considered to increase certainty that the resident is not infected. - If an observation area has been created, residents in the facility who develop symptoms consistent with COVID-19 could be moved from their rooms to this location while undergoing evaluation. - All recommended PPE should be worn during care of residents under observation; this includes use of an N95 or higher-level respirator (or facemask if a respirator is not available), eye protection (i.e., goggles or a disposable face shield that covers the front and sides of the face), gloves, and gown. Cloth face coverings are not considered PPE and should not be worn by HCP when PPE is indicated.   Evaluate and Manage Residents with Symptoms of COVID-19   - Ask residents to report if they feel feverish or have symptoms consistent with COVID-19. - Actively monitor all residents upon admission and at least daily for fever (T≥100.0 oF) and symptoms of COVID-19 (shortness of breath, new or change in cough, sore throat, muscle aches). If positive for fever or symptoms, implement Transmission-Based Precautions as described below. - Older adults with COVID-19 may not show typical symptoms such as fever or respiratory symptoms. Atypical symptoms may include new or worsening malaise, new dizziness, or diarrhea. Identification of these symptoms should prompt isolation and further evaluation for COVID-19. - The health department should be notified about residents or HCP with suspected or confirmed COVID-19, residents with severe respiratory infection resulting in hospitalization or death, or ≥ 3 residents or HCP with new-onset respiratory symptoms within 72 hours of each other. - Contact information for the healthcare-associated infections program in each state health department is available. - Information about the clinical presentation and course of patients with COVID-19 is described in the Interim Clinical Guidance for Management of Patients with Confirmed Coronavirus Disease 2019 (COVID-19). CDC has also developed guidance on Evaluating and Reporting Persons Under Investigation (PUI). - If COVID-19 is suspected, based on evaluation of the resident or prevalence of COVID-19 in the community, follow the Interim Infection Prevention and Control Recommendations for Patients with Suspected or Confirmed Coronavirus Disease 2019 (COVID-19) in Healthcare Settings. This guidance includes detailed information regarding recommended PPE, which includes use of an N95 or higher-level respirator (or facemask if a respirator is not available), eye protection (i.e., goggles or a disposable face shield that covers the front and sides of the face), gloves, and gown. Cloth face coverings are not considered PPE and should not be worn by HCP when PPE is indicated.   - Residents with suspected COVID-19 should be prioritized for testing. Residents with known or suspected COVID-19 do not need to be placed into an airborne infection isolation room (AIIR) but should ideally be placed in a private room with their own bathroom.   - Residents with COVID-19 should, ideally, be cared for in a dedicated unit or section of the facility with dedicated HCP (see section on Dedicating Space).   - As roommates of residents with COVID-19 might already be exposed, it is generally not recommended to place them with another roommate until 14 days after their exposure, assuming they have not developed symptoms or had a positive test.   - Increase monitoring of ill residents, including assessment of symptoms, vital signs, oxygen saturation via pulse oximetry, and respiratory exam, to at least 3 times daily to identify and quickly manage serious infection.   - Consider increasing monitoring of asymptomatic residents from daily to every shift to more rapidly detect any with new symptoms.   - If a resident requires a higher level of care or the facility cannot fully implement all recommended infection control precautions, the resident should be transferred to another facility that is capable of implementation. Transport personnel and the receiving facility should be notified about the suspected diagnosis prior to transfer.   - While awaiting transfer, residents should wear a cloth face covering or facemask (if tolerated) and be separated from others (e.g., kept in their room with the door closed)   - All recommended PPE should be used by healthcare personnel when coming in contact with the resident. - For decisions on removing residents with COVID-19 from Transmission-Based Precautions refer to the Interim Guidance for Discontinuation of Transmission-Based Precautions and Disposition of Hospitalized Patients with COVID-19   Additional Measures:   - Cancel communal dining and all group activities, such as internal and external activities. - Remind residents to practice social distancing and perform frequent hand hygiene. - Have residents wear a cloth face covering or facemask whenever they leave their room, including for procedures outside of the facility. - In addition to the actions described above, these are things facilities should do when there are COVID-19 cases in their facility or sustained transmission in the community:   - Healthcare Personnel Monitoring and Restrictions: Because of the higher risk of unrecognized infection among residents, universal use of all recommended PPE for the care of all residents on the affected unit (or facility-wide depending on the situation) is recommended when even a single case among residents or HCP is identified in the facility; this should also be considered when there is sustained transmission in the community. The health department can assist with decisions about testing of asymptomatic residents.   - Resident Monitoring and Restrictions: Encourage residents to remain in their room. If there are cases in the facility, restrict residents (to the extent possible) to their rooms except for medically necessary purposes. If they leave their room they should wear a cloth face covering or facemask, perform hand hygiene, limit their movement in the facility, and perform social distancing (stay at least 6 feet away from others) |
| ECRI, 2020a; Country: USA, Sponsor: ECRI  Scope: Infection control and prevention procedures and procedures during equipment servicing |
| ECRI has established the following levels of concern related to the risks of infection associated with the circumstances described:  No infection concern. There is no concern about infection from external surfaces that have been cleaned and disinfected.  Minimal infection concern. If the interior of a device is exposed to SARS-CoV from room air—most likely air drawn into the device by a cooling fan—there may be some concern about contamination. Until more is known about the transmission of SARS, we suggest that hospitals err on the side of caution and use simple protective measures for even such minimal-risk situations. These measures are readily implemented (see Specific Protective Steps, below). Note, however, that the warm air that is generally circulating inside a device with a cooling fan promotes drying and dilution of contaminants, which may reduce the viability of some viruses.  Higher infection concern. Surfaces that have been in contact with the patient's oral secretions or other excretions and cannot be readily disinfected pose a bigger concern. This is because, although the infection risk is still likely to be extremely low on these surfaces, there is a greater probability that the virus has remained viable. Such surfaces include the following:  Breathing circuits (including ventilator accessories and any portions of the breathing circuit inside ventilators), suction devices and systems, or any other devices that are exposed to the patient's oral secretions (including contaminated condensate), urine, feces, and other excretions  HEPA filtration systems, which are installed systems or portable systems (i.e., mobile high-efficiency-filter air cleaners [MHEFACs]) used to control room air contamination levels and ensure negative pressure in isolation rooms  Any handheld items or other items that can be found in beds, including nurse call buttons, remote controls for televisions, pillow speakers, blood pressure cuffs, and telemetry transmitters  Highest infection concern. The highest level of concern is posed by entering the room of a SARS patient without appropriate personal protective equipment (PPE). This is because of the close proximity to the patient, the potential risk of exposure to droplet secretions from coughs and sneezes, exposure to contaminated surfaces, and possible exposure associated with aerosol-generating procedures.  Keep in mind that even the highest level of risk is not an insurmountable concern as long as servicing personnel take the protective steps listed in the next section.  Specific Protective Steps  The following practices should be implemented to protect personnel while they are maintaining and repairing equipment that has been used on—or that has been in the same room as—patients who have or who are suspected of having SARS. For the most part, these represent good infection control practices that should be followed when servicing any device, independent of SARS concerns.  1. Do not enter the room of a SARS patient  Access to such rooms should be restricted to essential personnel only. This is to ensure the safety of personnel and to minimize disease transmission. However, if you must enter the room, follow relevant hospital procedures to minimize the exposure risks.  2. Minimize exposure of medical equipment to SARS  Before a patient with SARS is brought into a room, remove any unessential equipment. Use breathing-circuit filters to protect exhalation valves and other ventilation components from contamination (for more on this topic, see the Guidance Article Mechanical Ventilation of SARS Patients: Lessons from the 2003 SARS Outbreak). Use disposable devices or accessories for SARS patients whenever possible.  3. Observe proper hand hygiene  Frequent and thorough handwashing with soap and water is essential. Alcohol-based handrubs can be used when hands are not visibly soiled and handwashing facilities are not immediately available. Personnel should not rub their eyes or touch their mouth, nose, or other mucous membranes while working on exposed equipment. While wearing gloves, personnel should also avoid touching other surfaces in the room that are not involved in the equipment repair (e.g., doorknobs, telephones, test equipment, computer terminals, keyboards, manuals). In addition, personnel should not eat, drink, chew gum, smoke, or apply cosmetics until they have removed all protective wear and washed their hands.  4. Use proper decontamination and transport procedures  Equipment should not be transported until it has been cleaned and disinfected and disposables have been removed by housekeeping, central processing, or other appropriate personnel. (Note that commonly used disinfectants are effective against the SARS virus.) If equipment from the room of a SARS patient must be removed before the exterior can be cleaned and disinfected, follow any hospital policies on transporting contaminated devices.  5. Choose an appropriate work area  Equipment that poses particular infection concerns should be worked on in designated areas where servicing can be performed without the risk of infecting patients or other employees. These areas should not be near any patient care areas, food preparation or storage areas, medication areas, or other clean areas.  6. Wear protective equipment when appropriate  For personnel working on minimal-risk surfaces, we recommend using the following PPE:  Gloves  Clean, nonsterile gown, apron, or laboratory coat  Eye protection (e.g., goggles)  A face shield is an alternative form of eye protection. Although a face shield should provide adequate protection against an occasional minor splatter that may occur during servicing, the U.S. Occupational Safety and Health Administration (OSHA) requires the use of goggles (or special protective eyeglasses) when eye protection is used. CDC, on the other hand, recommends goggles or a face shield for protection against a splash or spray of body fluids.  Respiratory protection  Update: As of February 3, 2020, CDC recommends, in the case of the COVID-19 outbreak, that staff "use respiratory protection (i.e., a respirator) that is at least as protective as a fit-tested NIOSH-certified disposable N95 filtering facepiece respirator before entry into the patient room or care area." That recommendation matches the guidance ECRI Institute initially issued (in June 2003) in the case of the 2003 SARS outbreak.  Note, however, that respiratory protection recommendations can evolve over time. For example, as new information about airborne transmission of SARS became available and as concern lessened, we updated our recommendations for that circumstance: In February 2004, we specified that it would be prudent for personnel working with the equipment to wear a surgical mask, but that there was no significant benefit to using an N95 respirator for that application, particularly in light of the considerable time and effort that respirator use entails. (For additional information about respirator types and implementation challenges, see Selecting Respiratory Protection for Equipment Servicers and Other Hospital Personnel: Lessons from the 2003 SARS Outbreak.) It is unknown at this time if similar changes will develop in the case of the COVID-19 outbreak.  7. Before starting work . . .  If there is any question about whether the exterior surfaces of the equipment were adequately disinfected, including the bottom and back, disinfect those surfaces immediately. Also, if disposable components have not already been discarded, do that right away as well. If the equipment is not needed immediately, ECRI suggests allowing time—several hours to overnight—for viruses to die before servicing is carried out. (Note, however, that this waiting period should not be seen as a substitute for other infection control procedures.)  8. If the interior of the equipment is dusty . . .  Use a vacuum cleaner with a HEPA filter to remove dust as soon as adequate access is gained during disassembly and before working on the interior. Never blow on the equipment or use compressed air to remove dust or other particulates.  9. Clean up when done.  Clean and disinfect the work area after servicing is complete.  10. If an exposure occurs . . .  If you believe you have been exposed to SARS-CoV while unprotected, consult with the hospital's infection control practitioner, epidemiologist, or employee health staff for the procedures to follow. |
| ECRI, 2020b; Country: USA; Sponsor: ECRI  Scope: Advance preparation to limit the spread of infection |
| Recommendations for SARS that are also relevant to COVID-19   - Develop or improve the planning and decision-making structure for SARS detection and response. - If one is not already in place, develop a written SARS preparedness and response plan. - Assess the facility's ability to respond to SARS. - Establish an effective surveillance, triage, and clinical evaluation system. - Reinforce basic infection control practices. - Train staff to recognize potential SARS patients, know what actions to take when SARS is suspected, proficiently don and use personal protective equipment (PPE), and recognize and apply precautions during aerosol-generating procedures. - Reinforce the use of "respiratory hygiene/cough etiquette," including educating patients on respiratory hygiene (such as using hand-hygiene solutions and facial tissues and properly disposing of expended tissues) and providing surgical masks and/or tissues to patients to minimize droplet generation. - Use engineering controls (e.g., designated waiting rooms for patients with respiratory symptoms and/or Plexiglas barriers at the point of triage) and administrative controls and work practices (e.g., droplet precautions) to manage patients until the cause of their respiratory symptoms is determined. - Develop a patient transport and isolation plan. - Implement the proper design, operation, and maintenance of isolation rooms that will house SARS patients. - Implement a mechanism to report and evaluate exposures and apparent healthcare illness caused by SARS-CoV. - Have a strategy to meet increased staffing needs and clinical and protective equipment and supplies needs (e.g., ventilators for SARS patients) in the event of a SARS outbreak. - Develop strategies to limit access to the hospital. - Have a mechanism to ensure effective communication with public health departments and the public. - Establishment of adequate airborne infection isolation facilities. While the role of airborne transmission of SARS has not been fully established, CDC recommends that healthcare facilities admit patients with possible SARS to airborne infection isolation rooms (AIIRs) or specially adapted SARS units or wards, where patients can be safely managed. - Equipment procurement and training. Hospitals need to assess availability and anticipated need for consumable and durable medical equipment resources. Consumable supplies mentioned by CDC include hand hygiene supplies (antimicrobial soap and alcohol-based, waterless hand-hygiene products), disposable particulate respirators (N95 or higher), powered air-purifying respiratory (PAPR) hoods and battery packs (if applicable), goggles and face shields (disposable or reusable), gowns, gloves, and surgical masks. Durable equipment includes ventilators, portable high-efficiency particulate-air (HEPA) filtration units, and portable x-ray units - ECRI recommends that clinical engineering personnel assist in these preparations, especially for durable equipment, by doing the following: Locating and approving suppliers (preferably those suppliers that can reliably provide the models that staff are familiar with) and obtaining guarantees that supplies will be available; making sure that users receive training on models that are new to staff; developing procedures that ensure that rental or other temporary devices are logged in and are safe and functional before clinical use. Clinical engineering personnel should be prepared to conduct safety and functionality checks (including 24-hour on-call availability), should arrange with the device supplier (e.g., rental agency) to inspect devices according to an agreed-upon protocol, or should assist clinical staff in preparing an inspection protocol that they will use before using the devices. |
| Geffen, 2020; Country: South Africa; Sponsor: Samson Institute for Aging Research  Scope: infection prevention, testing and screening |
| - Environmental cleaning - All “touch-points” in the facility should be mapped out and these high touch areas included in a cleaning/de-sanitising checklist so that none are overlooked by cleaning staff - Treat every person as potentially infected with COVID-19 – this means that each resident’s room and laundry is assumed to be contaminated and treated with care - Conduct routine cleaning (ongoing and preventive) and terminal cleaning (when there is known contamination) - Terminal cleaning involves deep cleaning, combined with a disinfecting process. This can either be done as a two-step process, first using detergent and then disinfectant or a combined detergent/disinfectant product - Hard surfaces can be disinfected using hypochlorite solution (bleach e.g. JIK brand), alcohol (at least 60% concentration), hydrogen peroxide (3% concentration) or a hospital-grade disinfectant with activity against viruses. - Soft surfaces - should be laundered (see laundry section) - Electronics - such as keyboards, remote controls, touch screens, tablets and cell phones screens should be wiped down with alcohol-based wipes or sprays (70% alcohol concentration or more) and dried thoroughly. - Respiratory etiquette - Wearing of masks and face shields for source control - Cloth, surgical masks and face shields to help to prevent spread of respiratory secretions. - A universal mask wearing policy should apply - All staff to wear surgical masks at all times, regardless of whether there is COVID-19 in the facility. - Essential visitors should wear surgical or cloth masks at all times - Residents should be encouraged to wear masks, BUT masks should not be placed on anyone who has trouble breathing, or anyone who is unconscious, incapacitated, or otherwise unable to remove the mask without assistance. - Coughing and sneezing - All staff and residents must be regularly reminded to cover mouth and nose when coughing or sneezing – using a disposable tissue to cover if possible and then disposing immediately into a waste bin. - Make tissues and waste bins available throughout the facility - If this is not possible then individuals must sneeze into the crook of their elbow or sleeve. - Hand hygiene - Measures to put in place: - Liquid hand-soap and paper towels must be available at all hand basins - Alcohol-based rub stations must be available at the point of care, at the entrance to each resident’s room, in high-touch areas, in communal areas (residential and staff areas), eating spaces, and in all office spaces - Paper towel dispensers should be wall mounted close to the hand wash basin where soap dispensers are available - Training of all staff on hand washing and hand sanitising protocols - A mapping exercise of all high touch areas in the facility should be undertaken and hand sanitiser should be available in the close vicinity of all of these areas. - Signs should be placed throughout the facility in places where activities that necessitate hand washing or hand sanitising take place – e.g. dining areas, bathrooms, entrance/exit points to patient rooms, entrance to facility, care giver workspaces. - Hand hygiene education for staff, residents and education - Signs demonstrating appropriate hand washing and hand sanitising techniques should be placed at all wash sinks and hand sanitising stations in the facility. - Personal protection equipment (PPE) - All personnel should wear PPE while caring for vulnerable people - PPE will only prevent spread of infection if it is: - Used and changed at the right time - Accompanied by good hand hygiene - Cough etiquette is applied - Mobile phones should not be used whilst wearing PPE - All staff, including cleaners & housekeepers, must be trained in how to use PPE - Facemasks, eye protection, gowns, gloves should be available outside of residents rooms - A receptacle must be available near the exit of the room for single use equipment - A sign outside of the room describing precautions required and PPE needed - All staff with direct contact to older people should be wearing mouth-nose protection to protect patients, even when they are not engaging in direct care cases. - PPE Requirements: Overcoat/labcoat; Plastic poncho/apron; Mask (cloth/surgical/N95/KN95/FFP2); face shields; eye protection; gloves - Strategic use of PPE in resource-constrained settings to avoid shortages - Extended use of some PPE (respirators, facemasks, face shields, eye protection) for an entire shift is possible but care must be taken to avoid touching the PPE. If this happens hand hygiene must be performed immediately. - Face shields and goggles can be carefully decontaminated and re-used on another shift - Gowns/aprons should be prioritised for high-contact care or where splashes and sprays are anticipated. - Gowns/aprons should not be used for caring for different residents unless caring for a cohort of residents with COVID-19 infection and they do not have other known infections. - Managing consumables - In addition to the consumable products required for ongoing prevention, there should be adequate stocks of consumables needed for an outbreak and regular stock takes should be carried out for: - Cleaning materials and disinfectant products - Hand hygiene products (liquid soap, alcohol-based hand sanitiser, paper hand towels) - PPE for cleaning, care and health care staff - Residents - Residents should be encouraged to stay in their rooms or apartments as much as possible - Signs reminding residents to stay at least 2m from other residents in communal areas should be placed throughout the facility. - It may be difficult to expect residents living with dementia to maintain physical distancing behaviour. It may also be difficult to restrict the movement of residents living with dementia who “walk with intent” or wander. Wandering behaviour may also increase as their routines are disrupted by COVID-19 restrictions. - Employees - Employee areas - Includes offices and work stations, service areas, employee areas (entrances, tea room, toilets) - Carry out assessment of these areas and points of contact between people - Develop disinfection plan based on high-touch areas, especially computer equipment - Space desks and other work stations to facilitate physical distancing (2m apart) - Place hand sanitiser at all work stations and desks - Provide alcohol wipes or spray to staff members to wipe down personal and communal computer equipment and devices. - Place floor markings to encourage physical distancing - Require mask wearing - Keep areas well ventilated - Discourage the sharing of stationery - Staff shifts - Place employees in teams/shifts to minimise contact with other teams or shifts - Don’t move employees between teams or shifts - Stagger lunch and tea breaks to enable social distancing - If there is space, appropriate facilities and agreement from care workers, having care workers stay in the facility for their shifts to minimise their community exposure and need to travel on public transport should be considered. - Staff should not be allowed to work in multiple facilities and should rather be brought into one facility full-time - Managing entry to the facility and residents’ rooms - Employee protocol: - Care workers should sign in at the door to the facility and perform hand hygiene immediately - Daily COVID-19 screening and temperature checks to be performed at the door to the facility (see Screening Stations in Section 2). - All employees should be made aware of COVID-19 symptoms and should be instructed to phone the facility for screening if they are showing any symptoms or feeling unwell in any way. - Employees should take special care while using public transport - Care workers should not travel to work in their work uniforms and should change immediately after arriving. - All care workers should only work at one care facility - Non-punitive pay policies should be developed for quarantine/isolation or COVID-related sick-leave to discourage employees from concealing sickness. - Health visitors - Where possible, all health consults should be performed using telehealth services - Where this is not possible the following measures must be in place: - Temperature checks and COVID-19 screening of all health visitors (see Screening Stations in Section 2) - Registration of all health visitors to facilitate contact tracing - The wearing of masks and following facility hand hygiene protocol is required - Friends and family - Lockdowns for residents can have unintended consequences which include: increase in staff workload, loneliness, boredom, loss of physical and cognitive function, increase in behaviours suggesting unmet needs and an increase in chemical and physical restraint. - However, the possibility of allowing essential visitors needs to be weighed up against the virus reproduction rate and community prevalence. - In the case of HIGH RISK OF COMMUNITY TRANSMISSION, a no visitor policy should be strictly enforced, except in circumstances where a resident is gravely ill. In these cases, family or persons providing emotional care should be allowed on compassionate grounds. Examples of compassionate grounds include:   - Resident is nearing end of life   - To support residents with dementia or other mental illness who are distressed   - Screening and risk assessment at building entrance for staff and visitors - Setup of screening area - All staff and any essential visitors should be screened daily for COVID-19 symptoms. - A screening station should be established at the facility entrance and staff entrance (if separate). - A non-contact infrared thermometer should be available for temperature checks. - This should be held 15cm from the entrant’s forehead, temple or neck - Any temperature over 38 degrees Celsius is considered a fever - Each entrant who passes the temperature check should fill in their details and should be asked to note if they are experiencing any of the listed symptom. - The register of those entering each day should be filed at the end of each day and a new sheet used for the following day. This will allow for easier contact tracing should there be an outbreak in the facility. - The screening station should have pens available in two containers marked “New” and “Used”. Pens should be taken from the new cup and used pens should be sanitised using an alcohol wipe. - Screening process - Any entrant should first be temperature checked at the door. - Those entering the building should sanitise hands before approaching the screening station - Each person should complete the spreadsheet - The individual managing the screening station should check their questions - Staff co-morbidity screening/risk assessment and testing procedures - Risk Assessment - It is important to understand whether any staff are at significant risk due to co-morbidities or age - Provisions should be made for admin staff with underlying chronic conditions or who are over the age of 60 to work from home if possible. - All staff should complete a confidential health screener which determines the presence of significant underlying conditions - Care workers or health staff with the above underlying conditions who are likely to have close contact with residents, should not be expected to work with confirmed COVID-positive residents. - Testing procedures for staff - Testing procedure followed will vary in accordance with government testing policy at the time. - If there are large backlogs in public testing, symptom monitoring should be employed and testing only conducted if an individual is likely to have had significant close contact with vulnerable individuals without PPE - Symptom tracking for staff - Persons who have had contact with persons under investigation or COVID-positive individuals should track their symptoms daily - Those in quarantine should be called daily and their symptoms recorded in a spreadsheet - Staff being actively monitored can be tracked via regular staff entry protocol but should be aware of both common and more atypical symptoms and should be instructed to call in to report any emerging symptoms rather than arriving to work. - Symptom tracking can also be carried out using a publicly available app which will also provide staff with further advice and instructions, but this data is stored in a central repository and may not be available to the facility. (https://app.testforcovid.co.za/) - Screening and testing of residents - All residents should be screened daily for COVID-19 symptoms and have their temperature checked. - Screening and temperature checks should be conducted by nursing staff on duty - Thermometer must be sterilised between uses on residents - Screening of persons with dementia - Communication with persons with dementia may be challenging and may make screening for COVID-19 more difficult among residents with dementia - It is important that carers LOOK for signs if COVID-19 rather than rely on reported symptoms • People with dementia may have swallowing difficulties which could put them at increased risk of developing chest infections and dehydration – a swallowing assessment may be helpful. - Testing of suspected cases or exposed persons - All residents who are symptomatic should be isolated and tested - If a resident is exposed to a staff member or other resident who has developed symptoms or tested positive, the resident should be quarantined and monitored closely for the emergence of symptoms. - Management of staff showing symptoms or testing positive for COVID-19 - Tracing contacts - If a staff member or residents becomes ill, it is essential to trace all close contact within the facility (both staff and residents) so that quarantine and isolation processes can be put in place as quickly as possible. - Those who have had close contact with a COVID-19 case or PUI without appropriate PPE should be entered onto a contact tracing sheet and treated in accordance with the table in Section 3. - Identifying source of infection - It is important to establish whether the exposure occurred at work or in a community setting - All exposed staff members should be interviewed to determine how the exposure occurred. It should be made clear that the interview is not meant for disciplinary purposes, but in order to prevent future exposures. - In cases where staff appear to have been infected at work, interview all exposed staff to improve systems / training on use of PPE and other protocols to prevent future cases within the facility and identify any other staff and residents that may not have been considered during contact tracing. - Testing procedures   - If staff or residents become infected or are placed under investigation, those with close contact with the infected persons should be traced using staffing logs to determine which other staff or residents the PUI or infected person has had close contact with.   - See testing procedures for staff under Screening and Testing   Quarantine and isolation procedures   - Quarantine of staff - Asymptomatic or pre-symptomatic people with close contact with a COVID-positive individual or PUI that have developed symptoms and are awaiting test results should quarantine for 14 days. - Ideally, staff should isolate at home, but if this is not possible, they must go to a quarantine facility. - Ideally, even people with negative test results, but who show COVID-19 symptoms should still go into quarantine for 14 days. - In the case of essential health care workers, testing can be conducted after 8 days and if negative the health care worker can resume work sooner - Isolation of staff - COVID-positive individuals must isolate for 14 days from exposure and can either self- isolate at home or be admitted to a dedicated isolation facility. - Ideally individuals who do not require hospitalisation should isolate at home, but if this is not possible, they must go to a facility. - Management of residents under investigation and confirmed cases - Tracing contacts - If a resident becomes ill, it is essential to trace all close contacts (both staff and residents) within the facility so that quarantine and isolation processes can be put in place as quickly as possible. - Those who have had close contact with a COVID-19 case or PUI without appropriate PPE should be entered onto a contact tracing sheet and treated in accordance with the table in Section 3. - If a resident has had contact with visitors, contact tracing must also be carried out in relation to their families and other persons they may have come into close contact with, but this will be carried out by the DoH rather than the facility.   Quarantine and isolation of residents   - Quarantine timeframes - Ideally, even people with negative test results but who show COVID-19 symptoms should still remain in quarantine for 14 days. - Individuals must quarantine for 14 days from exposure - Residents in quarantine should be housed in individual rooms to avoid infecting others or being infected by COVID-positive individuals (some PUIs will be positive while others may not be) - Rooms should be marked to alert staff to their status and residents should receive no visitors, wear a mask when interacting with care workers or health staff and appropriate PPE needs to be worn by any entering the room - Masks should be worn by both the person in quarantine and others providing care for or in contact with the quarantined person. - In the case of an outbreak, it is not necessary to set up a quarantine section of the facility as re-locating residents is likely to spread the virus through the facility. - Limit the number of staff members entering the room to essential staff and ideally allocate one care worker to the individual per shift and one additional care worker who may be called upon for activities that require two persons (e.g. lifting). - Care workers in close contact with isolated residents should not work with residents in the general population. - Follow all infection and prevention control precautions outlined in Section 1, including use of PPE, enforcement of strict protocol in relation cleaning, disinfection and disposal of medical and other waste" |
| HPS, 2020; Country: United Kingdom (Scotland); Sponsor: Health Protection Scotland  Scope: support those working in care home settings to give advice to their staff and users of their services about COVID-19 |
| Preventing spread of infection in Care Home Settings  Hand hygiene  Hand hygiene is essential to reduce the transmission of infection in health and other care settings. All staff, residents and visitors should decontaminate their hands with soap and water or alcohol-based hand rub (ABHR) when entering and leaving areas where patient care is being delivered. See Appendix 2 for best practice on hand washing.  Hand hygiene must be performed immediately before every episode of direct patient care and after any activity or contact that potentially results in hands becoming contaminated, including the removal of personal protective equipment (PPE), equipment decontamination and waste handling  Respiratory and cough hygiene – ‘Catch it, bin it, kill it’  Residents, staff and visitors should be encouraged to minimise potential COVID-19 transmission through good respiratory hygiene measures which are:   disposable, single-use tissues should be used to cover the nose and mouth when sneezing, coughing or wiping and blowing the nose – used tissues should be disposed of promptly in the nearest waste bin   tissues, waste bins (lined and foot operated) and hand hygiene facilities should be available for patients, visitors and staff   hands should be cleaned (using soap and water if possible, otherwise using ABHR) after coughing, sneezing, using tissues or after any contact with respiratory secretions and contaminated objects   encourage patients to keep hands away from the eyes, mouth and nose  Some patients (such as the elderly and children) may need assistance with containment of respiratory secretions; those who are immobile will need a container (for example a plastic bag) readily at hand for immediate disposal of tissues.  Providing care for residents during COVID-19 pandemic  Ensure daily monitoring of all individuals for COVID-19 symptoms, or other signs of illness. Residents with cognitive impairment may be less able to report symptoms. Elderly or frail residents with co-morbidities may also present with atypical and non-specific signs of illness. Further information can accessed here. If a resident becomes unwell contact the GP for clinical advice  A single case of infection should prompt contact with your local HPT as it may also signal the start of a possible outbreak. An outbreak is normally defined as two linked cases of a disease. For care homes specifically, with respect to COVID-19, an outbreak should be suspected when there is a single new case with symptoms consistent with COVID-19 infection arising in the care home, likely to be due to spread of the virus within the care home  On identification of a new suspected COVID-19 case, you must contact your local HPT without delay who will advise on the need for testing of residents and staff as well as other IPC measures to help limit further spread of the virus and control the outbreak  Testing in the care home  Currently all care home residents and staff who develop symptoms should be clinically assessed and where appropriate offered testing for COVID-19.  Resident placement  All symptomatic or COVID-19 diagnosed residents in the facility should be isolated immediately for 14 days from the date of symptom onset (or date of first positive test if symptom onset undetermined). The individual should be placed in a single room with en-suite facilities, where possible. The door should be kept closed to the room. Where this is not possible, ensure the bed is moved to the furthest safe point in the room to achieve a 2 metre physical distance to the open door. Clearly sign the rooms by placing IPC signs, indicating droplet precautions, at the entrance of the room or area. Confidentiality must be maintained  Cohorting of symptomatic individuals:  Cohorting in care homes should be avoided where possible. Individuals who are shielding (extremely high risk) must not be placed in cohorts and should be prioritised for single occupancy rooms. Where all single isolation room facilities are occupied and cohorting is unavoidable, then cohorting can be arranged so that  Confirmed COVID-19 individuals are placed in multi-occupancy rooms together.  Suspected COVID-19 individuals are placed in multi occupancy rooms together.  Confirmed and suspected cases should not be cohorted together  Personal Protective Equipment (PPE)  PPE used for sessional use  Aprons and gloves are subject to single use as per Standard Infection Control Precautions (SICPs), and must be changed between residents. Respirators, fluid-resistant (Type IIR) surgical masks (FRSM), eye protection and disposable fluid repellent coveralls or long-sleeved disposable fluid repellent gowns can be subject to sessional use in circumstances outlined in the PPE tables  Environmental decontamination (cleaning and disinfection)  Those carrying out the cleaning must be familiar with the required environmental and equipment decontamination processes, be trained in these accordingly and ensure they are wearing the appropriate PPE. People responsible for cleaning should be advised to clean the COVID-19 areas and isolation room(s) after all other unaffected areas of the facility have been cleaned.  COVID-19 affected areas should be cleaned at least twice daily paying particular attention to common touch surfaces such as door handles, tablets, mobile phones, light switches, remote controls and bed rails  Therefore, decontamination of the environment should be performed as per Appendix 5 using either  A combined detergent disinfectant solution at a dilution of 1000 parts per million available chlorine (ppm available chlorine (av.cl.));  or  A detergent clean followed by disinfection (1000ppm av.cl.).  Environmental decontamination where the suspected or confirmed case is no longer in the room/ environment  The immediate area occupied by the individual and any equipment used by the individual, should be cleaned using the methods described above. Once this process has been completed, the area can be put back into use.  Staffing  15.1 Staff Cohorting (working in dedicated teams)  Assigning a dedicated team of staff to care for individuals with COVID-19 is an additional IPC measure which can help prevent onward spread of infection. This should be implemented whenever there are sufficient levels of staff available (so as not to have a negative impact on non-affected individual care).  15.2 Minimise external staff  The use of bank or agency staff should be minimised. Where used then they should only work for one facility where possible.  Contractors on site should be kept to a minimum and only essential work carried out.  15.3 Ensure staff are enabled to follow key measures described in this guidance to prevent spread  Ensure that all individuals in the facility are aware of the requirement to self-isolate if they or their household members develop symptoms of COVID-19 or are diagnosed with COVID-19 and support them in doing so.  Follow Test and Protect advice on NHS Inform where appropriate  Consider the additional demands that will be placed on people by requirements for household isolation and put in place resilience planning to support this. |
| Lester, 2020; Country: USA; Sponsor: Not applicable  Scope: policies for skilled nursing facilities to prepare for and manage COVID-19 |
| "Measures Regarding Staff  Screen all employees when reporting for duty for fever, symptoms of respiratory illness, and other COVID-19 symptoms. Do not let anyone enter if they have fever or symptoms of COVID-19. Screener should be wearing a surgical mask.  If your community might participate in contact tracing, then a written sign-in log should be maintained for anyone who enters the facility.  Provide a face mask daily to each staff member to be worn at all times while in the facility. This mask should be available at the front entrance, prior to contact with the screener. The screener should be stationed at least 6 feet away from the area of those entering the facility.  Staff should have a place to eat meals that allows them to practice appropriate social distancing while eating without masks.  Usage of locker rooms should follow social distancing guidelines while protecting employees’ rights.  The following are mandatory once COVID-19 is known to be in the facility, are strongly recommended if COVID-19 is becoming prevalent in your community, and should be strongly considered if equipment is available regardless of local COVID-19 prevalence.  Screening Measures for Residents/Patients  Screen all residents for COVID symptoms along with measurements of temperature and pulse oximetry at least twice daily. The facility medical director should set criteria for a positive screen.  The SNF should be prioritized for rapid, point of care testing as it is the bestway to manage the epidemic in real time. Until this is available, facilities should be provided with a sufficient supply of test kits for PCR testing to meet diagnostic needs of the facility on an ongoing basis with access to a laboratory that can provide results of PCR testing within 24 hours.  Avoid group activities (such as recreational activities and physical and occupational therapy) that do not allow for the maintenance of 6 feet social distancing. Notably, it is often difficult for ambulatory residents with dementia to follow social distancing rules.  Because COVID-19 can spread prior to detection, to minimize risk of spread, convert nebulizer medications to metered dose inhalers (MDI) and stop nasal sprays which might spread virus  Communication  Consider use of Telehealth visits for Medical consultant providers (dermatology, podiatry, etc) for use when necessary with proper cleaning of this equipment.  Arrange for video or window visits between residents and families.  Provide regular updates on the status of COVID-19 in the facility to staff. This can be though written, e-mail or video updates, and can improve morale.  Develop ID cards with prominent photo of staff with name and title, to help residents identify caregivers who are wearing PPE obscuring the face.  Develop a color coding system for doors regarding COVID-19 status to remind staff to use appropriate PPE.  Provide in-service to staff regarding proper use of PPE and hand washing, and post signs as reminders.  Recognize that many older adults have hearing impairment. Many will have difficulty understanding healthcare providers wearing masks, which muffles sound as well as eliminates ability to lip read. Consider basic communication boards in each room to ask residents questions in writing.  The facility should develop a protocol to notify other patients and families residing in that facility regarding COVID status per local Department of Health regulations. Possible ways to achieve this include updating the facility website daily to inform families or utilizing a robo-call system.  Inform residents directly (if cognitively aware) and family members/designated representatives about diagnosis of COVID-19. Share your treatment plan and discuss advance directives.  Plan for a memorial/remembrance service following social distancing guidelines when acute management of the crisis has resolved sufficiently to allow for reflection and shared condolences.  Admissions/Re-admissions  The authors do not support the mandatory admission of patients with COVID-19 from hospitals to nursing homes as it may force unprepared facilities to provide care to patients with COVID without the necessary resources or precautions.  Hospitalized patients who are known COVID-19 positive should be admitted to a COVID positive unit.  If space allows, hospitalized patients who are COVID-19 negative, or were not tested, can be admitted to a “transition” unit for 14 days while they are monitored for symptoms of COVID-19 and tested if indicated (and available).  Additional supportive measures for staff and residents:  Unlike hospital staff who generally care for patients for short periods of time, the SNF staff care for SNF residents often for many years. This strong connection can make the death of SNF residents even more devastating. Emotional support should be provided to staff as they grieve loss of residents.  Cheerful drawings and messages from the community can be uplifting to SNF workers and patients. They can be posted in hallways and distributed to residents.  Many hospitals are touting their “success” stories as patients coming off a ventilator or being discharged. “Success” in the SNF, especially for long-term care residents, is different." |
| MOH, 2020; Country: Canada; Sponsor: Ministry of Health  Scope: screening, infection control for COVID-19 in long-term care |
| Screening  Passive screening:   - As part of routine measures for the respiratory season, signage should be visible and remind all persons in the LTCH to perform hand hygiene and follow respiratory etiquette. - Signage should indicate signs and symptoms of COVID-19 and steps that must be taken if COVID-19 is suspected or confirmed in a staff member or a resident.   Active screening for staff:   - LTCHs should instruct all staff to self-monitor for COVID-19 at home. All persons should be made aware of signs and symptoms of COVID-19 infection, as listed in the COVID-19 Provincial Testing Guidance Update document. - LTCHs must conduct active screening for COVID-19 symptoms of all staff, essential visitors, and anyone else entering the home. Screening must include twice daily (at the beginning and end of the day or shift) symptom screening, including temperature checks. This excludes emergency first responders - Essential visitors include a person performing essential support services (e.g., food delivery, maintenance, family providing care services, and other health care) or a person visiting a very ill or palliative resident. If an essential visitor is admitted to the home, precautions must be taken as outlined in Directive #3 for Long-Term Care Homes under the Long-Term Care Homes Act, 2007. - LTCHs should have a screener at the entrance who is able to conduct screening during business hours and change of shift. Outside of these times, the home’s charge nurse/administrator should develop processes and procedures to ensure that all persons entering the home are screened and visits are logged. These procedures are to be applied seven days a week and 24 hours a day.   Active screening for residents:   - LTCHs should conduct active screening of all residents, at least twice daily (at the beginning and end of the day) to identify if any resident has symptoms of COVID-19, including temperature checks. Residents with symptoms (including mild respiratory symptoms or atypical symptoms) must be isolated and tested for COVID-19.   Active Screening for Resident Admissions, Resident Re-Admissions:   - LTCHs should screen new admissions and re-admissions for symptoms and potential exposure to COVID-19. All new residents must be placed in isolation under contact and droplet precautions upon admission to the home and tested within 14 days of admission. If test results are negative, the resident must remain in isolation for 14 days from arrival. If test results are positive, refer to the Testing for COVID-19 section below. - Hospitals are being asked by the ministry to temporarily stop transfers to long-term care and retirement homes. However, in the unlikely event that a transfer is still required, patients transferred from a hospital to a long-term care home or retirement home must be tested, and results received, prior to transfer. A negative result does not rule out the potential for incubating illness and all patients should remain under droplet and contact precautions for a 14-day isolation period following transfer. - For information regarding new admissions and re-admissions during an outbreak, please refer to the Outbreak Guidance for Long-Term Care Homes document as well as the Control of Respiratory Infection Outbreaks in Long-Term Care Homes document.   Positive Screening: What to do   - Anyone showing symptoms of COVID-19 should not be allowed to enter the LTCH and should go home immediately to self-isolate. - Residents with symptoms of COVID-19 must be isolated in droplet and contact precautions and tested. - Staff should provide care to residents with suspect or confirmed COVID-19 using the precautions outlined in Directive #1 for Health Care Providers and Health Care Entities, as well as Public Health Ontario’s Technical Brief on IPAC Recommendations for Use of Personal Protective Equipment for Care of Individuals with Suspect or Confirmed COVID-19.   Summary of Required Precautions  Preventing spread from staff or essential visitors who may be asymptomatic/pre-symptomatic while working in the LTCH or visiting the LTCH:   - Use a surgical/procedure mask at all times during shift or visit. - For staff who are taking breaks, the surgical/procedure mask may be removed but a minimum two metre distance should be maintained from others.   Before providing care to a resident:   - Staff must conduct a point-of-care risk assessment to determine the precautions required.   Providing care to residents with suspect or confirmed COVID-19, including collection of nasopharyngeal and oropharyngeal swabs:   - Droplet and Contact Precautions, including:   - Surgical/procedure mask   - Isolation gown   - Gloves   - Eye protection (goggles/face shield)   Providing CPAP and/or open suctioning to resident with suspect or confirmed COVID-19:   - Droplet and Contact precautions plus use of N95 respirator. - Manage in single room with door closed. - Keep the number of people in the room during the procedure to a minimum.   Testing for COVID-19   - LTCHs should implement a very low threshold for COVID-19 testing. Testing must be conducted on every symptomatic resident and staff member in the LTCH as outlined in the COVID-19 Provincial Testing Guidance Update document. - LTCHs must consider a single, laboratory confirmed case of COVID-19 in a resident or staff member as a confirmed COVID-19 outbreak in the LTCH. In a new admission or re-admission who tests positive, it may not be necessary to declare an outbreak if they have been in isolation under contact and droplet precautions since entering the LTCH. Outbreaks should be declared in collaboration between the home and health unit to ensure an outbreak number is provided. - LTCHs that are testing patients for COVID-19 should review PHO’s guidelines for testing including Specimen Collection and Handling procedures, and how to prepare samples prior to transport. - For information regarding testing during an outbreak, please refer to the Outbreak Guidance for Long-Term Care Homes document and the Control of Respiratory Infection Outbreaks in Long-Term Care Homes document.   Reporting of Positive Screening   - COVID-19 is a designated disease of public health significance (O. Reg. 135/18) and thus reportable under the Health Protection and Promotion Act. - The LTCH should contact their local public health unit to report a staff member or resident suspected to have COVID-19. The local public health unit will provide specific advice on what control measures should be implemented to prevent further spread and how to monitor for other possible infected residents and staff members. LTCHs must also follow the critical incident reporting requirements in section 107 of O. Reg 79/10 under the Long-Term Care Homes Act. - All referrals to hospital should be made through emergency department triage. If a resident is referred to a hospital, the LTCH should coordinate with the hospital, local public health unit, paramedic services, and the resident to ensure safe travel that maintains the resident in appropriate isolation precautions. Patient transfer services should not be used to transfer a resident with suspect or confirmed COVID-19.   Occupational Health & Safety  Staff Exposure/Staff Illness:   - All staff who have been advised to self-monitor for 14 days from an exposure should discuss with their supervisor. - All staff who are required to self-isolate must not come to work. Anyone with symptoms compatible with COVID-19 must not come to work, must get tested, and must report their symptoms to the LTCH. Staff responsible for occupational health at the LTCH must follow up on all staff who have been advised to self-isolate. For details on work self-isolation please see COVID-19 Outbreak Guidance for Long-Term Care Homes (LTCH). - Staff who test positive for COVID-19 should report their illness to their manager/supervisor or to Employee Health/Occupational Health and Safety as per usual practice. The manager/supervisor or Employee Health/Occupational Health designate must promptly inform the Infection Control Practitioner or designate of any cases or clusters of staff including contract staff who are absent from work. - If COVID-19 is suspected or diagnosed in a staff, return to work should be determined in consultation with their health care provider and the local public health unit. Staff must report to Occupational Health and Safety prior to return to work. Detailed general occupational health and safety guidelines for COVID-19 are available on the MOH COVID-19 website.   Personal Protective Equipment:   - LTCHs must following the precautions outlined in Directive #1 for Health Care Providers and Health Care Entities.   Mask Use for Source Control:   - LTCHs should immediately implement that all staff and essential visitors wear a surgical/procedure mask at all times for the duration of full shifts or visits in the LTCH. Staff may remove their surgical/procedure mask during breaks but must remain at least two metres away from others to prevent any potential transmission of COVID-19. LTCHs should have written procedures, instruction, and training for staff on mask use (e.g. how to wear and remove a mask).   Limiting Work Locations:   - Wherever possible, LTCH employers should work with staff, contractors, and volunteers to limit the number of work locations that staff, contractors, and volunteers are working, to minimize risk to residents and other staff of exposure to COVID-19. - LTCH employers must also comply with Ontario Regulation 146/20 made pursuant to the Emergency Management and Civil Protection Act.   Environmental Cleaning:   - Patient-contact surfaces (i.e., areas within 2 metres of the person who has screened positive) should be disinfected as soon as possible (refer to PIDAC Routine Practices and Additional Precautions in All Health Care Settings for more information about environmental cleaning). |
| WHO, 2020; Country: Not Applicable; Sponsor: World Health Organization  Scope: prevent COVID-19 from entering the facility, prevent COVID-19 from spreading within the facility, and prevent COVID-19 from spreading to outside the facility |
| "System and service coordination to provide long-term care  Coordinate with relevant authorities (e.g. Ministry of Health, Ministry of Social Welfare, Ministry of Social Justice, etc.) should be in place to provide continuous care in LTCFs.  Activate the local health and social care network to facilitate continuous care (clinic, acute-care hospital, day-care center, volunteer group, etc.).  Facilitate additional support (resources, health care providers) if any older person in LTCFs is confirmed with COVID19  IPC focal point and activities  LTCFs should ensure that there is an IPC focal point at the facility to lead and coordinate IPC activities, ideally supported by an IPC team with delegated responsibilities and advised by a multidisciplinary committee.  the IPC focal point should:  Provide COVID-19 IPC training 1to all employees, including: an overview of COVID-19 (https://openwho.org); hand hygiene and respiratory etiquette; standard precautions; and COVID-19 transmission-based precautions  Provide information sessions for residents on COVID-19 to inform them about the virus, the disease it causes and how to protect themselves from infection;  Regularly audit IPC practices (hand hygiene compliance) and provide feedback to employees.  Increase emphasis on hand hygiene and respiratory etiquette  Maintain high standards of hygiene and sanitation practice.  Provide annual influenza vaccination and pneumococcal conjugate vaccines to employees and staff, according to local policies, as these infections are important contributors to respiratory mortality in older people.  Physical distancing in the facility  Physical distancing in the facility should be instituted to reduce the spread of COVID-19:  Restrict the number of visitors (see below)  For group activities ensure physical distancing, if not feasible cancel group activities.  Stagger meals to ensure physical distance maintained between residents or if not feasible, close dining halls and serve residents individual meals in their rooms.  Enforce a minimum of 1 meter distance between residents.  Require residents and employees to avoid touching (e.g., shaking hands, hugging, or kissing).  Visitors  In areas where COVID-19 transmission has been documented, access to visitors in the LTCFs should be restricted and avoided as much as possible. Alternatives to in-person visiting should be explored, including the use of telephones or video, or the use of plastic or glass barriers between residents and visitors.  All visitors should be screened for signs and symptoms of acute respiratory infection or significant risk for COVID-19 (see screening, above), and no one with signs or symptoms should be allowed to enter the premises.  Direct contact by visitors with residents with confirmed or suspected COVID-19 should be prohibited.  Early recognition  Early identification, isolation and care of COVID-19 cases is essential to limit the spread of the disease in the LTCFs.  Prospective surveillance for COVID-19 among residents and staff should be established:  Assess health status of any new residents at admission to determine if the resident has signs of a respiratory illness including fever2 and cough or shortness of breath.  Assess each resident twice daily for the development of a fever (≥38C), cough or shortness of breath.  Immediately report residents with fever or respiratory symptoms to the IPC focal point and to clinical staff.  Prospective surveillance for employees should be established:  Ask employees to report and stay at home if they have fever or any respiratory illness.  Follow up on employees with unexplained absences to determine their health status.  Undertake temperature check for all employees at facility entrance.  Immediately remove from service any employee who is visibly ill at work and refer them to their health care provider.  Monitor employees and their contact with residents, especially those with COVID-19; use the WHO risk assessment tool to identify employees who have been at high risk of exposure to COVID-19.  Prospective surveillance for visitors should be established:  All visitors should be screened before being allowed to see residents, including for fever, respiratory illness and if they have had recent contact with someone infected with COVID-19.  Visitors with fever or any respiratory illness should be denied access to the facility.  Visitors with significant risk factors for COVID-19 (close contact to a confirmed case, recent travel to an area with community transmission [applies only to those areas that do not have current community transmission] should be denied access to the facility.  Source control (care for the COVID-19 patient and prevention of onward transmission)  Precautions and personal protective equipment (PPE)  When providing routine care for a resident with suspected or confirmed COVID-19, contact precaution and droplet precautions should be practiced. Detailed instructions on precautions for COVID-19 are available.  PPE should be put on and removed carefully following recommended procedures to avoid contamination.  Hand hygiene should always be performed before putting on and after removing PPE.  Contact and droplet precautions include the following PPE: medical mask, gloves, gown, and eye protection (goggles or face shield).  Employees should take off PPE just before leaving a resident’s room.  Discard PPE in medical waste bin and preform hand hygiene.  Environmental cleaning and disinfection  Hospital-grade cleaning and disinfecting agents are recommended for all horizontal and frequently touched surfaces (e.g., light switches, door handles, bed rails, bed tables, phones) and bathrooms being cleaned at least twice daily and when soiled.  Visibly dirty surfaces should first be cleaned with a detergent (commercially prepared or soap and water) and then a hospital-grade disinfectant should be applied, according to manufacturers’ recommendations for volume and contact time. After the contact time has passed, the disinfectant may be rinsed with clean water.  If commercially prepared hospital-grade disinfectants are not available, the LTCFs may use a diluted concentration of bleach to disinfect the environment. The minimum concentration of chlorine should be 5000 ppm or 0.5% (equivalent to a 1:9 dilution of 5% concentrated liquid bleach)  Laundry  Soiled linen should be placed in clearly labelled, leak-proof bags or containers, after carefully removing any solid excrement and putting it in a covered bucket to be disposed of in a toilet or latrine.  Machine washing with warm water at 60−90°C (140−194°F) with laundry detergent is recommended. The laundry can then be dried according to routine procedures.  If machine washing is not possible, linens can be soaked in hot water and soap in a large drum using a stick to stir and being careful to avoid splashing. The drum should then be emptied, and the linens soaked in 0.05% (500 ppm) chlorine for approximately 30 minutes. Finally, the laundry should be rinsed with clean water and the linens allowed to dry fully in sunlight.  Restriction of movement/ transport  Confirmed patients should not leave their rooms while ill.  Restrict movement or transport of residents to essential diagnostic and therapeutic tests only.  Avoid transfer to other facilities (unless medically indicated)  Reporting  Any suspected or confirmed COVID-19 cases should be reported to relevant authorities as required by law or mandate." |

# APPENDIX 7 – CPG Coding Summary and Supporting Text

| Category | Supporting Text |
| --- | --- |
| Cohorting equipment | Choose an appropriate work area: Equipment that poses particular infection concerns should be worked on in designated areas where servicing can be performed without the risk of infecting patients or other employees |
| Communication | Have a mechanism to ensure effective communication with public health departments and the public |
|  | Coordinate with relevant authorities (e.g. Ministry of Health, Ministry of Social Welfare, Ministry of Social Justice, etc.) should be in place to provide continuous care in LTCFs; Any suspected or confirmed COVID-19 cases should be reported to relevant authorities as required by law or mandate |
|  | A single case of infection should prompt contact with your local HPT as it may also signal the start of a possible outbreak. |
|  | Provide regular updates on the status of COVID-19 in the facility to staff. This can be though written, e-mail or video updates, and can improve morale. |
| Consulting/notifying health professionals | If an exposure occurs If you believe you have been exposed to SARS-CoV while unprotected, consult with the hospital's infection control practitioner, epidemiologist, or employee health staff for the procedures to follow. |
|  | If COVID-19 is suspected or diagnosed in a staff, return to work should be determined in consultation with their health care provider and the local public health unit. |
|  | coordination with several important stakeholders such as Geriatrics health professionals, NH leadership teams (e.g., administrators, medical directors, and directors of nursing), Hospice and palliative care experts, and Local collaborations |
| Diagnostic testing | LTCHs should implement a very low threshold for COVID-19 testing. Testing must be conducted on every symptomatic resident and staff member |
| Disinfecting Surfaces | Use proper decontamination and transport procedures |
|  | Patient-contact surfaces (i.e., areas within 2 metres of the person who has screened positive) should be disinfected as soon as possible |
|  | Before starting work If there is any question about whether the exterior surfaces of the equipment were adequately disinfected, including the bottom and back, disinfect those surfaces immediately. Also, if disposable components have not already been discarded, do that right away as well. If the equipment is not needed immediately, ECRI suggests allowing time—several hours to overnight—for viruses to die before servicing is carried out. (Note, however, that this waiting period should not be seen as a substitute for other infection control procedures.) |
|  | If the interior of the equipment is dusty Use a vacuum cleaner with a HEPA filter to remove dust as soon as adequate access is gained during disassembly and before working on the interior |
|  | Clean up when done (Clean and disinfect the work area after servicing is complete.) |
|  | Develop a schedule for regular cleaning and disinfection of shared equipment, frequently touched surfaces in resident rooms and common areas; Ensure EPA-registered, hospital-grade disinfectants are available to allow for frequent cleaning of high-touch surfaces and shared resident care equipment. |
|  | All “touch-points” in the facility should be mapped out and these high touch areas included in a cleaning/de-sanitising checklist so that none are overlooked by cleaning staff |
|  | Hospital-grade cleaning and disinfecting agents are recommended for all horizontal and frequently touched surfaces (e.g., light switches, door handles, bed rails, bed tables, phones) and bathrooms being cleaned at least twice daily and when soiled |
|  | Those carrying out the cleaning must be familiar with the required environmental and equipment decontamination processes, be trained in these accordingly and ensure they are wearing the appropriate PPE. People responsible for cleaning should be advised to clean the COVID-19 areas and isolation room(s) after all other unaffected areas of the facility have been cleaned. COVID-19 affected areas should be cleaned at least twice daily paying particular attention to common touch surfaces such as door handles, tablets, mobile phones, light switches, remote controls and bed rails |
| Droplet precautions | Providing CPAP and/or open suctioning to resident with suspect or confirmed COVID-19: Droplet and Contact precautions plus use of N95 respirator. |
|  | When providing routine care for a resident with suspected or confirmed COVID-19, contact precaution and droplet precautions should be practiced. |
| Education | Educate Residents, Healthcare Personnel, and Visitors |
|  | All NH staff caring for residents who test positive for COVID-19 should be trained in infection control, the use of PPE, and recognition of COVID-19 symptoms |
|  | Train staff to recognize potential SARS patients, know what actions to take when SARS is suspected, proficiently don and use personal protective equipment (PPE), and recognize and apply precautions during aerosol-generating procedures |
|  | Provide COVID-19 IPC training 1to all employees, including: an overview of COVID-19 (https://openwho.org); hand hygiene and respiratory etiquette; standard precautions; and COVID-19 transmission-based precautions; Provide information sessions for residents on COVID-19 to inform them about the virus, the disease it causes and how to protect themselves from infection |
| Hand hygiene | Reinforce adherence to standard infection prevention and control measures including hand hygiene |
|  | As part of routine measures for the respiratory season, signage should be visible and remind all persons in the LTCH to perform hand hygiene and follow respiratory etiquette |
|  | Observe proper hand hygiene |
|  | Liquid hand-soap and paper towels must be available at all hand basins; Alcohol-based rub stations must be available at the point of care; Training of all staff on hand washing and hand sanitising protocols |
|  | Hand hygiene is essential to reduce the transmission of infection in health and other care settings. All staff, residents and visitors should decontaminate their hands with soap and water or alcohol-based hand rub (ABHR) when entering and leaving areas where patient care is being delivered |
| Policies for Visitors | Visitor Restrictions |
|  | Consider New Policies and Procedures for Visitors |
|  | Policies and Procedures for Visitors |
| Personal Protective Equipment | Wear protective equipment when appropriate |
|  | Use a surgical/procedure mask at all times during shift or visit; Providing care to residents with suspect or confirmed COVID-19, including collection of nasopharyngeal and oropharyngeal swabs, droplet and Contact Precautions, including: Surgical/procedure mask; Isolation gown; Gloves; Eye protection (goggles/face shield) |
|  | All recommended PPE should be worn during care of residents under observation; this includes use of an N95 or higher-level respirator (or facemask if a respirator is not available), eye protection (i.e., goggles or a disposable face shield that covers the front and sides of the face), gloves, and gown |
|  | All personnel should wear PPE while caring for vulnerable people; PPE will only prevent spread of infection if it is used and changed at the right time; Mobile phones should not be used whilst wearing PPE; All staff, including cleaners & housekeepers, must be trained in how to use PPE |
|  | PPE should be put on and removed carefully following recommended procedures to avoid contamination; Hand hygiene should always be performed before putting on and after removing PPE. |
|  | Aprons and gloves are subject to single use as per Standard Infection Control Precautions (SICPs), and must be changed between residents. Respirators, fluid-resistant (Type IIR) surgical masks (FRSM), eye protection and disposable fluid repellent coveralls or long-sleeved disposable fluid repellent gowns can be subject to sessional use in circumstances outlined in the PPE tables |
|  | Provide a face mask daily to each staff member to be worn at all times while in the facility. This mask should be available at the front entrance, prior to contact with the screener. |
| Policies for visitors | LTCHs must conduct active screening for COVID-19 symptoms of all staff, essential visitors, and anyone else entering the home; Anyone showing symptoms of COVID-19 should not be allowed to enter the LTCH and should go home immediately to self-isolate |
|  | LTCHs should immediately implement that all staff and essential visitors wear a surgical/procedure mask at all times for the duration of full shifts or visits in the LTCH |
|  | any essential visitors should be screened daily for COVID-19 symptoms; Registration of all health visitors to facilitate contact tracing; The wearing of masks and following facility hand hygiene protocol is required |
| Provide supplies | Provide Supplies for Recommended Infection Prevention and Control Practices; assess current PPE supply and implement strategies to optimize current supply |
|  | [provide] masks, face shields, gowns, and gloves that all frontline healthcare professionals and direct care workers need in order to protect themselves against becoming infected |
|  | Supplies for diagnostic and serologic testing are integral to protecting the health and safety of all Americans during a pandemic |
|  | Have a strategy to meet increased staffing needs and clinical and protective equipment and supplies need |
|  | Strategic use of PPE in resource-constrained settings to avoid shortages; Extended use of some PPE (respirators, facemasks, face shields, eye protection) for an entire shift is possible but care must be taken to avoid touching the PPE. If this happens hand hygiene must be performed immediately |
| Respiratory hygiene/cough etiquette | Reinforce the use of "respiratory hygiene/cough etiquette," including educating patients on respiratory hygiene (such as using hand-hygiene solutions and facial tissues and properly disposing of expended tissues) and providing surgical masks and/or tissues to patients to minimize droplet generation |
|  | As part of routine measures for the respiratory season, signage should be visible and remind all persons in the LTCH to perform hand hygiene and follow respiratory etiquette |
|  | Respiratory Hygiene and Cough Etiquette: Tissues and trash cans are available in common areas and resident rooms for respiratory hygiene and cough etiquette and source control |
|  | All staff and residents must be regularly reminded to cover mouth and nose when coughing or sneezing – using a disposable tissue to cover if possible and then disposing immediately into a waste bin. |
|  | Respiratory and cough hygiene – ‘Catch it, bin it, kill it’; Residents, staff and visitors should be encouraged to minimise potential COVID-19 transmission through good respiratory hygiene measures which are: disposable, single-use tissues should be used to cover the nose and mouth when sneezing, coughing or wiping and blowing the nose – used tissues should be disposed of promptly in the nearest waste bin; tissues, waste bins (lined and foot operated) and hand hygiene facilities should be available for patients, visitors and staff |
| Social distancing/ isolation/cohorting | Individuals who test positive for COVID-19 should not be discharged to a mainstream NH unless the facility can safely and effectively isolate the patient from other residents |
|  | Minimize group activities inside the facility or field trips outside of the facility. |
|  | Develop criteria for halting group activities and communal dining, closing units or the entire facility to new admissions, and restricting visitation. |
|  | Create a plan for cohorting residents with symptoms of respiratory infection, including dedicating HCP to work only on affected units. |
|  | Do not enter the room of a SARS patient |
|  | Residents should be encouraged to stay in their rooms or apartments as much as possible; Signs reminding residents to stay at least 2m from other residents in communal areas should be placed throughout the facility; Space desks and other work stations to facilitate physical distancing (2m apart); Place employees in teams/shifts to minimise contact with other teams or shifts; Residents quarantined in LTCFs should be in individual rooms, rooms should be marked to alert staff, residents should receive no visitors, wear a mask when interacting with care givers. |
|  | Physical distancing in the facility should be instituted to reduce the spread of COVID-19: Restrict the number of visitors (see below); For group activities ensure physical distancing, if not feasible cancel group activities; Stagger meals to ensure physical distance maintained between residents or if not feasible, close dining halls and serve residents individual meals in their rooms; Enforce a minimum of 1 meter distance between residents; Require residents and employees to avoid touching (e.g., shaking hands, hugging, or kissing). |
|  | Cohorting in care homes should be avoided where possible. Individuals who are shielding (extremely high risk) must not be placed in cohorts and should be prioritised for single occupancy rooms. Where all single isolation room facilities are occupied and cohorting is unavoidable, then cohorting can be arranged so that: Confirmed COVID-19 individuals are placed in multi-occupancy rooms together; Suspected COVID-19 individuals are placed in multi occupancy rooms together; Confirmed and suspected cases should not be cohorted together |
|  | Assigning a dedicated team of staff to care for individuals with COVID-19 is an additional IPC measure which can help prevent onward spread of infection |
|  | Avoid group activities (such as recreational activities and physical and occupational therapy) that do not allow for the maintenance of 6 feet social distancing. |
| Staffing Policies | Reinforce sick leave policies; remind HCP not to report to work when ill |
|  | Paid leave and assistance for frontline healthcare workers |
|  | Wherever possible, LTCH employers should work with staff, contractors, and volunteers to limit the number of work locations that staff, contractors, and volunteers are working, to minimize risk to residents and other staff of exposure to COVID-19. |
|  | Staff should not be allowed to work in multiple facilities and should rather be brought into one facility full-time; Non-punitive pay policies should be developed for quarantine/isolation or COVID-related sick-leave to discourage employees from concealing sickness. |
|  | The use of bank or agency staff should be minimised. Where used then they should only work for one facility where possible. Contractors on site should be kept to a minimum and only essential work carried out. |
| Surveillance/monitoring/evaluating | Evaluate and Manage HCP with Symptoms of Respiratory Illness |
|  | Evaluate and Manage Residents with Symptoms of Respiratory Infection |
|  | Healthcare Personnel Monitoring and Restrictions |
|  | Resident Monitoring and Restrictions |
|  | Screen all HCP at the beginning of their shift for fever and symptoms of COVID-19 |
|  | NHs should implement policies and procedures for screening staff aligned with guidance from the CDC and updated regularly to account for situational change |
|  | Establish an effective surveillance, triage, and clinical evaluation system. |
|  | LTCHs must conduct active screening for COVID-19 symptoms of all staff, essential visitors, and anyone else entering the home. |
|  | All staff and any essential visitors should be screened daily for COVID-19 symptoms.; A screening station should be established at the facility entrance and staff entrance (if separate).; A non-contact infrared thermometer should be available for temperature checks.; All residents should be screened daily for COVID-19 symptoms and have their temperature checked. |
|  | Prospective surveillance for COVID-19 among residents and staff should be established: Assess health status of any new residents at admission to determine if the resident has signs of a respiratory illness including fever2 and cough or shortness of breath.; Assess each resident twice daily for the development of a fever (≥38C), cough or shortness of breath.; Immediately report residents with fever or respiratory symptoms to the IPC focal point and to clinical staff. |
|  | Ensure daily monitoring of all individuals for COVID-19 symptoms, or other signs of illness. Residents with cognitive impairment may be less able to report symptoms. |
|  | Screen all employees when reporting for duty for fever, symptoms of respiratory illness, and other COVID-19 symptoms. Do not let anyone enter if they have fever or symptoms of COVID-19. Screener should be wearing a surgical mask; Screen all residents for COVID symptoms along with measurements of temperature and pulse oximetry at least twice daily. The facility medical director should set criteria for a positive screen. |
